# Species of *Ergasilus* von Nordmann, 1832 (Copepoda: Ergasilidae) from cichlid fishes in Lake Tanganyika

**DOI:** 10.1017/S0031182023000239

**Published:** 2023-06

**Authors:** Robert Míč, Eva Řehulková, Mária Seifertová

**Affiliations:** Department of Botany and Zoology, Faculty of Science, Masaryk University, Kotlářská 2, 611 37, Brno, Czech Republic

**Keywords:** Africa, cichlids, diversity, parasitic crustaceans, Tanganyika

## Abstract

*Ergasilus* (von Nordmann, 1832) (Ergasilidae) is a species-rich group of parasitic copepods with a wide distribution in freshwater, marine and brackish environments. Up to now, 9 species of *Ergasilus* are known from cichlid fishes in Africa. In this study, 5 species, including 3 new, were collected from the gills of 12 cichlid species (11 genera: *Bathybates*, *Ctenochromis*, *Eretmodus*, *Gnathochromis*, *Lamprologus*, *Neolamprologus*, *Ophthalmotilapia*, *Perissodus*, *Simochromis*, *Spathodus* and *Tanganicodus*) of the northeastern shore of Lake Tanganyika in Burundi, namely *E. macrodactylus* (Sars, 1909), *E. megacheir* (Sars, 1909), *E*. *caparti* n. sp., *E*. *parasarsi* n. sp. and *E. parvus* n. sp. All species found were identified and described on the basis of adult female specimens using an integrative taxonomy approach mixing morphological characterization and molecular analyses of 2 ribosomal DNA markers (partial 18S and 28S rDNA sequences). An identification key for *Ergasilus* species from Lake Tanganyika is included. This study provides the first molecular data for *Ergasilus* species in Africa. The phylogenetic analyses suggest that the *Ergasilus* species parasitizing Lake Tanganyikan cichlids form a well-supported clade within the Ergasilidae. However, their phylogenetic relationships with other congeners still remain unclear due to a lack of molecular data for this diverse genus.

## Graphical abstract

**Figure:**
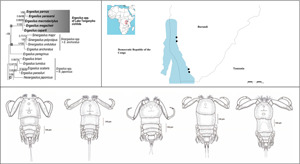

## Introduction

Lake Tanganyika, one of the largest (32 900 km^2^) tropical lakes and the oldest (~9–12 Ma) of the East African Great Lakes (Cohen *et al*., 1993), represents a unique freshwater ecosystem characterized by high levels of species richness and a high degree of endemism. Therefore, it has received an intensive interest from scientists of various fields for decades (e.g. Kmentová *et al*., 2018; Rahmouni *et al*., 2018; Koblmüller *et al*., 2019; Ryan *et al*., 2020; Ivory *et al*., 2021). In this respect, cichlids (Cichlidae) have become one of the most studied model systems in the research on evolutionary processes, speciation events (e.g. Hayward *et al*., 2017; Irisarri *et al*., 2018; Fischer *et al*., 2021) and behavioural biology (e.g. Raffini *et al*., 2018; Satoh *et al*., 2019, 2022). Currently, 240 valid cichlid species belonging to 52 genera and 13 tribes are described from Lake Tanganyika; all (except 2 species) are endemic to the basin (Ronco *et al*., 2021) and comprise the morphologically, behaviourally and ecologically most diverse species assemblage (Snoeks, 2000). It is not surprising then that cichlid fishes have also become a suitable model for studies of host–parasite systems. In recent years, the most studied fish parasites from Lake Tanganyika have been, in particular, Monogenea (e.g. Vanhove *et al*., 2015; Rahmouni *et al*., 2017, 2018), in contrast to parasitic crustaceans that have been neglected for decades, not only in Lake Tanganyika but in Africa overall (Scholz *et al*., 2018).

*Ergasilus* (von Nordmann, 1832) represents the type and most speciose genus of the family Ergasilidae (Burmeister, 1835), with approximately 160 nominal species worldwide, inhabiting fresh, marine and brackish waters (Boxshall and Defaye, 2007; WoRMS Editorial Board, 2022). In recent years, the majority of systematic studies dealing with ergasilid species originated from the Neotropical region (Marques *et al*., 2015; Muriel-Hoyos *et al*., 2015; Taborda *et al*., 2016; Varella *et al*., 2019; Narciso *et al*., 2020; Santacruz *et al*., 2020; Waicheim *et al*., 2021), which is in contrast to Africa, where no new records have been available in the past few decades. The first studies of African Ergasilidae were conducted between 1900 and 1928 in the African Great Lakes (Sars, 1909; van Douwe, 1912; Cunnington, 1920; Gurney, 1928), while the first description of *Ergasilus* species from Africa was made by Sars (1909), who described 3 species (originally assigned to the currently invalid genus *Ergasiloides*) from Lake Tanganyika. From the 1920s to the late 1960s, the next occurrences of *Ergasilus* species were reported in large African river systems such as the Niger River (Capart, 1956), the Volta (Paperna, 1969), the Congo (Fryer, 1963, 1967), the White Nile (Wilson, 1924) and the lakes (Albert, Edward, Kivu, Malawi, Rudolf, Tanganyika and Victoria) (Fryer, 1956, 1960, 1961, 1965). To date, 11 valid species of *Ergasilus* are described from the gills of 13 families of freshwater fishes from Africa, 5 of them recorded on cichlids, mochokids and poeciliids from Lake Tanganyika: *E. flaccidus* Fryer, 1965; *E. kandti* van Douwe, 1912; *E. macrodactylus* (Sars, 1909); *E. megacheir* (Sars, 1909); and *E. sarsi* Capart, 1944 (Sars, 1909; Cunnington, 1914; Capart, 1944; Fryer, 1965; Oldewage and van As, 1988; Kilian and Avenant-Oldewage, 2013; Smit and Hadfield, 2018). However, since Fryer's research (1965, 1968), records of ergasilids from Lake Tanganyika are scarce. Kondo and Hori (1986) and Raeymaekers *et al*. (2013) reported unidentified species of the genus *Ergasilus* in the southern end of Lake Tanganyika, but no morphological determination was provided in either of the studies. The last study that included ergasilids from Tanganyika was by Kilian and Avenant-Oldewage (2013) that reported the first record of *E. sarsi* from Tanganyika killifish and provides a description of the pathological alterations caused by this species. Nevertheless, over the last 30 years, the interest in Ergasilidae in Africa has declined. This suggests that the ergasilid fauna in Africa is still largely underexplored and that its intensive investigation is therefore needed.

Moreover, all previous records of *Ergasilus* species are based solely on morphometric data. Until recently, the majority of available molecular data for *Ergasilus* came from Song *et al*. (2008), who provided the first phylogenetic analysis of the family Ergasilidae (comprising 14 species collected in China) and proposed a polyphyletic origin for this genus. Molecular data for the African ergasilids are still completely lacking. Such data would help to resolve the taxonomical and phylogenetic relationships poorly known in this highly diverse parasitic group.

During our parasitological survey of metazoan parasites of cichlids from Lake Tanganyika, we recovered 2 previously described and 3 new *Ergasilus* species. All species found were described using a combined morphological and molecular approach as an integrative taxonomy, a recent trend that promises to complete dubious and precise descriptions and other taxonomic problems. Additionally, the phylogenetic relationships among ergasilids from Lake Tanganyika cichlids were investigated on the basis of rDNA sequence data (partial 18S and 28S rDNA).

## Materials and methods

### Fish collection

**Fig. 1.**
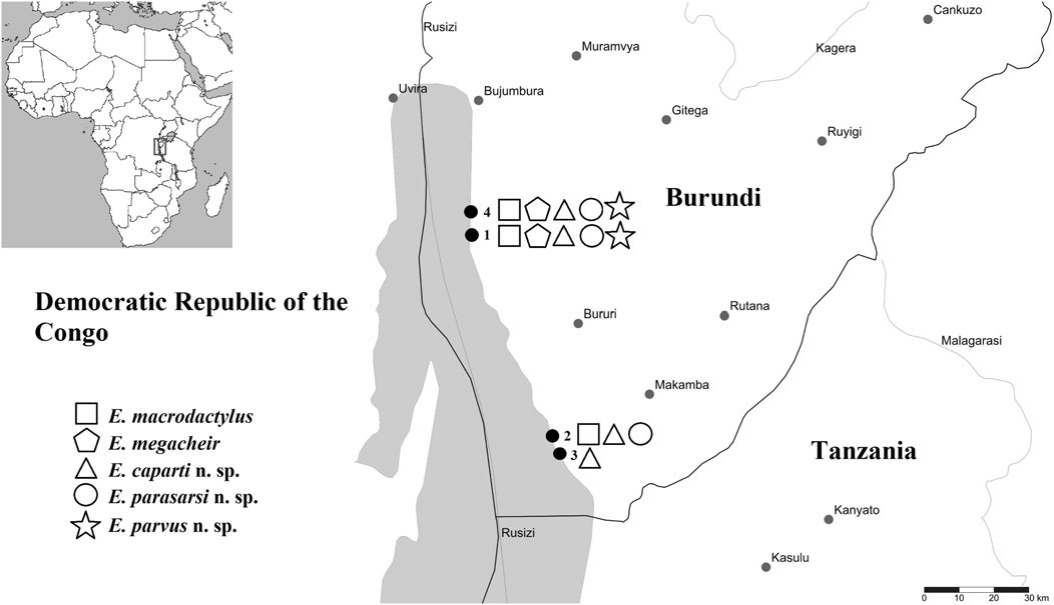
Map of Lake Tanganyika indicating the sampling localities along the northeastern shore in Burundi: (1) Magara; (2) Mukuruka; (3) Mvugo; (4) Nyaruhongoka.

During a parasitological survey in 2013, 23 species (167 specimens) of cichlid fishes, representing 11 tribes, were bought from a local fish market in Bujumbura (3°23′S, 29°22′E) or obtained from commercial fishermen fishing in 4 localities on the northeastern shore of Lake Tanganyika in Burundi: (1) Magara (3°44′S, 29°19′E); (2) Mukuruka (4°14′S, 29°33′E), (3) Mvugo (4°15′S, 29°34′E) and (4) Nyaruhongoka (3°41′S, 29°20′E) (Fig. 1, Table 1). The determination of cichlid hosts was provided by Dr Stephan Koblmüller (The University of Graz, Austria) using the keys of Takahashi (2003) and Takahashi and Koblmüller (2011). Live fish specimens were killed by severing the spinal cord and were subsequently processed for parasitological examination. The scientific names of fish hosts follow Froese and Pauly (2022).

**Table 1.** List of all sampled fishes and their sampling localities

Host species	Locality name and number	Coordinates
*Astatotilapia burtoni* (Günther, 1894)	Fish market Bujumbura	3°23′S, 29°22′E
*Aulonocranus dewindti* (Boulenger, 1899)	Magara, Lake Tanganyika, Burundi (1)	3°44′S, 29°19′E
***Bathybates ferox* (Boulenger, 1898)**	Nyaruhongoka, Lake Tanganyika, Burundi (4)	3°41′S, 29°20′E
*Bathybates graueri* (Steindachner, 1911)	Fish market Bujumbura*	3°23′S, 29°22′E
*Bathybates minor* (Boulenger, 1906)	Fish market Bujumbura	3°23′S, 29°22′E
*Benthochromis tricoti* (Poll, 1948)	Fish market Bujumbura	3°23′S, 29°22′E
*Boulengerochromis microlepis* (Boulenger, 1899)	Fish market Bujumbura	3°23′S, 29°22′E
*Cyprichromis microlepidotus* (Poll, 1956)	Fish market Bujumbura	3°23′S, 29°22′E
	Nyaruhongoka, Lake Tanganyika, Burundi (4)	3°41′S, 29°20′E
***Ctenochromis horei* (Günther, 1894)**	Fish market Bujumbura	3°23′S, 29°22′E
	Magara, Lake Tanganyika, Burundi (1)	3°44′S, 29°19′E
	Mvugo, Lake Tanganyika, Burundi (3)	4°15′S, 29°34′E
	Nyaruhongoka, Lake Tanganyika, Burundi (4)*	3°41′S, 29°20′E
***Eretmodus marksmithi* (Burgess, 2012)**	Magara, Lake Tanganyika, Burundi (1)*	3°44′S, 29°19′E
	Mukuruka, Lake Tanganyika, Burundi (2)*	4°14′S, 29°33′E
	Nyaruhongoka, Lake Tanganyika, Burundi (4)*	3°41′S, 29°20′E
***Gnathochromis permaxillaris* (David, 1936)**	Mukuruka, Lake Tanganyika, Burundi (2)*	4°14′S, 29°33′E
*Gnathochromis pfefferi* (Boulenger, 1898)	Mvugo, Lake Tanganyika, Burundi (3)	4°15′S, 29°34′E
*Hemibates stenosoma* (Boulenger, 1901)	Fish market Bujumbura	3°23′S, 29°22′E
***Lamprologus callipterus* (Boulenger, 1906)**	Magara, Lake Tanganyika, Burundi (1)*	3°44′S, 29°19′E
*Limnochromis auratus* (Boulenger, 1901)	Mukuruka, Lake Tanganyika, Burundi (2)*	4°14′S, 29°33′E
	Nyaruhongoka, Lake Tanganyika, Burundi (4)*	3°41′S, 29°20′E
	Fish market Bujumbura	3°23′S, 29°22′E
***Neolamprologus brichardi* (Poll, 1974)**	Magara, Lake Tanganyika, Burundi (1)*	3°44′S, 29°19′E
	Nyaruhongoka, Lake Tanganyika, Burundi (4)	3°41′S, 29°20′E
***Neolamprologus mondabu* (Boulenger, 1906)**	Mvugo, Lake Tanganyika, Burundi (3)*	4°15′S, 29°34′E
	Nyaruhongoka, Lake Tanganyika, Burundi (4)*	3°41′S, 29°20′E
***Ophthalmotilapia nasuta* (Poll & Matthes, 1962)**	Magara, Lake Tanganyika, Burundi (1)*	3°44′S, 29°19′E
***Perissodus microlepis* (Boulenger, 1898)**	Magara, Lake Tanganyika, Burundi (1)*	3°44′S, 29°19′E
	Nyaruhongoka, Lake Tanganyika, Burundi (4)*	3°41′S, 29°20′E
***Simochromis diagramma* (Günther, 1893)**	Magara, Lake Tanganyika, Burundi (1)*	3°44′S, 29°19′E
	Nyaruhongoka, Lake Tanganyika, Burundi (4)	3°41′S, 29°20′E
***Spathodus erythrodon* (Boulenger, 1900)**	Magara, Lake Tanganyika, Burundi (1)*	3°44′S, 29°19′E
***Tanganicodus irsacae* (Poll, 1950)**	Mukuruka, Lake Tanganyika, Burundi (2)*	4°14′S, 29°33′E
*Trematocara unimaculatum* (Boulenger, 1901)	Fish market Bujumbura	3°23′S, 29°22′E

Parasitized fishes are highlighted in bold, localities on which the host was parasitized by *Ergasilus* is marked with the symbol*.

### Parasite collection and identification

The body surface, gills and nasal cavities of freshly killed fishes were examined for the presence of parasitic copepods using a dissecting microscope. Live copepods were collected from the gills using fine needles and were fixed in 70% ethanol for later morphological examination, or in 96% ethanol for molecular analysis. In addition, a subset of specimens collected for DNA analyses were dissected and the egg sac or a small part of the body were cut off using fine needles under a dissecting microscope and used for DNA extraction; the rest of the body was used for morphological evaluation as hologenophore. For the morphological determination of copepods, specimens were placed on a microscopic slide in a drop of water with lactic acid to clear and soften them. The selected crustaceans were then fixed in pure glycerine (Dávidová and Smit, 2018) or glycerine ammonium picrate and examined. The mounted specimens were studied using an Olympus BX61 microscope equipped with phase-contrast optics. Drawings of the copepods were made using an Olympus drawing attachment and edited with a graphic tablet (Wacom Intuos5 Touch) compatible with Adobe Illustrator and Adobe Photoshop (Adobe Systems Inc., San Jose, CA, USA). All measurements (in micrometres) were taken using a digital image analysis software (Olympus Stream Motion v. 1.9.3) and are presented as mean followed by range and number (*n*) of specimens measured in parentheses. *Ergasilus* species were determined according to Oldewage and van As (1988) on the basis of the shape and size of the body, the antennae, the antennules, the cephalothorax, pigmentation, the setae and spines on legs I–IV, rudimentary leg V, the genital somite, the furcal rami and the shape of the egg sac. Morphological terminology follows that of Huys and Boxshall (1991).

For comparative purposes, specimens of the following 7 previously described species of *Ergasilus* from the Natural History Museum (London, UK; BMNH) and Royal Belgian Institute of Natural Sciences (Bruxelles, Belgium; RBINS COP) were examined:

*E. cunningtoni* Capart, 1944 (BMNH 1950.7.29.23); *E. flaccidus* Fryer, 1965 (BMNH 1965.10.6.1); *E. kandti* Douwe, 1912 (BMNH 1998.929-930); *E. lamellifer* Fryer, 1961 (BMNH 1998.931); *E. latus* Fryer, 1960 (BMNH 1993.122-131); *E. macrodactylus* (Sars, 1909) (COP 6086, COP 6087, COP 6088, COP 6089); and *E. sarsi* Capart, 1944 (COP 0493, COP 0494).

The type and voucher specimens of the copepods collected in the present study were deposited in the Institute of Parasitology, Czech Academy of Sciences, České Budějovice, Czech Republic. Prevalence (percentage of infected fish) and mean intensity of infection (mean number of parasites per infected host) were calculated for each *Ergasilus* species found following Bush *et al*. (1997).

### Molecular and phylogenetic analyses

Genomic DNA was isolated separately from each parasite specimen (or its part) using DNeasy®Blood & Tissue Kit (Qiagen, Hilden, Germany) according to the manufacturer's instructions. To perform molecular characterization of the species found and to elucidate their phylogenetic position within Ergasilidae, 2 ribosomal nuclear fragments (18S rDNA and 28S rDNA) were analysed. Partial 28S rDNA fragment was amplified using primers 28SF (forward, 5′-ACA ACT GTG ATG CCC TTA G-3′) and 28SR (reverse, 5′-TGG TCC GTG TTT CAA GAC G-3′) (Song *et al*., 2008). Primers 18SF (forward, 5′-AAG GTG TGM CCT ATC AAC T-3′) (Song *et al*., 2008) and 18SR (reverse, 5′-TTA CTT CCT CTA AAC GCT C-3′) (Song *et al*., 2008) were used for amplification of the partial fragment of 18S rDNA. In addition, the newly designed primers Erg18SF1 (forward, 5′-ATT GGA GGG CAA GTC TGG TG-3′), Erg18SF2-int (forward, 5′-CGA TCA GAT ACC GCC CTA GT-3′) and Erg18SR2 (reverse, 5′-AAG GGC AGG GAC GTA ATC AA-3′) were used for amplifications of the 18S rDNA fragment when the first combination of primers failed. All PCRs were carried out in a total volume of 20 *μ*L containing 3 *μ*L of DNA extract, 1× PCR buffer (Fermentas, Waltham, MA, USA), 2 mm MgCl_2_, 200 *μ*m of each dNTP, 0.2 *μ*m of each primer and 1 U of Taq polymerase (Fermentas). Amplification was performed under the following conditions: 94°C for 5 min; 39 cycles of 94°C for 30 s; an annealing temperature of 54°C for 30 s; and 72°C for 1 min, with a final extension step at 72°C for 5 min. The PCR amplicons were checked by electrophoresis on 1.5% agarose gels stained with GoodView™ (Amplia s.r.o., Bratislava, Slovakia), and PCR products of the required length were purified using ExoSAP-IT™ (Affymetrix Inc., Santa Clara, USA), following the manufacturer's instructions. Purified products were directly sequenced using the same primers as those for PCR. DNA sequencing was carried out using BigDye® Terminator v3.1 Cycle Sequencing Kit (Applied Biosystems by Thermo Fisher Scientific, Prague, Czech Republic) and a 3130 Genetic Analyzer (Applied Biosystems). The obtained sequences were assembled and edited using Sequencher software (Gene Codes Corp., Ann Arbor, MI, USA). Newly generated sequences for each species were deposited in GenBank under accession numbers OQ407470–OQ407474 (28S rDNA) and OQ407465–OQ407469 (18S rDNA), and molecular vouchers (hologenophores, paragenophores; Pleijel *et al*., 2008) were deposited in the Institute of Parasitology, Czech Academy of Sciences, České Budějovice, Czech Republic.

To investigate the phylogenetic position of copepods of Lake Tanganyika cichlids, relevant available sequences of Ergasilidae from 5 genera were retrieved from GenBank (for details, see Table 2). Three species of the family Lernaeidae, *Lernaea cyprinacea* (Linnaeus, 1758), *Lamproglena chinensis* (Yü, 1937) and *Lamproglena orientalis* (Markevich, 1936) were selected as outgroup. Partial sequences of 18S rDNA and 28S rDNA were aligned separately using MAFFT v.7 (Katoh and Standley, 2013), applying the G-INS-i iterative refinement algorithm. Gaps and ambiguously aligned regions were removed from the alignments with Gblocks v0.91b (Talavera and Castresana, 2007) using settings for a less stringent selection. jModelTest 2.1.10 (Darriba *et al*., 2012) was employed to select the most appropriate model of DNA evolution, using the Bayesian information criterion for each individual gene. The most suitable evolutionary model was TIM3e + I + G for the partial gene encoding 18S rRNA and TIM3 + F + I + G for the partial gene of 28S rRNA. The final phylogenetic reconstruction was performed on the concatenated dataset including partial 18S rDNA (991 bp) and partial 28S rDNA (588 bp) using maximum likelihood (ML) and Bayesian inference (BI) methods. ML analyses were carried out using IQ-TREE (Nguyen *et al*., 2015) on the W-IQ-TREE webserver (Trifinopoulos *et al*., 2016) and nodal support for the tree was assessed through ultrafast bootstrap approximation with 1000 replicates (Hoang *et al*., 2018). BI analysis was run in MrBayes 3.2.6 (Huelsenbeck and Ronquist, 2001) using the CIPRES platform (Miller *et al*., 2010), by setting the GTR + F + I (nst = 6 rates = invgamma) model for each partition; the analysis included 2 simultaneous runs of Markov chain Monte Carlo for 10 000 generations, sampling every 100 generations, with a ‘burn-in’ of 25%. The results were checked in Tracer v. 1.7.1 (Rambaut *et al*., 2018) to assess chain convergence. The trees were visualized and edited in FigTree v1.4.3 (Rambaut, 2012). Genetic distances (uncorrected *p*-distance) were calculated in MEGA v.11 (Tamura *et al*., 2021).

**Table 2.** List of parasitic copepods used for phylogenetic analysis, including their host species, collection locality and accession numbers for partial 18S and 28S rDNA sequences

Parasite species	Host species	Host family	Locality	GenBank accession numbers		Reference
				18S	28S	
Ergasilidae						
*Acusicola margulisae*	*Amphilophus citrinellus*	Cichlidae	Nicaragua	MN852694	MN852851	Santacruz *et al*. (2020)
*Ergasilus anchoratus*	*Tachysurus fulvidraco*	Bagridae	Baoan Lake, China	DQ107564	DQ107528	Song *et al*. (2008)
*Ergasilus briani*	*Misgurnus anguillicaudatus*	Cobitidae	Dangjiangkou, China	DQ107572	DQ107532	Song *et al*. (2008)
***Ergasilus caparti* n. sp.**	*Neolamprologus brichardi*	Cichlidae	Lake Tanganyika, Burundi	OQ407469	OQ407474	present study
*Ergasilus hypomesi*	*Acanthogobius hasta*	Gobiidae	Dangjiangkou, China	DQ107573	DQ107539	Song *et al*. (2008)
***Ergasilus macrodactylus***	*Gnathochromis permaxillaris*	Cichlidae	Lake Tanganyika, Burundi	OQ407465	OQ407470	present study
***Ergasilus megacheir***	*Simochromis diagramma*	Cichlidae	Lake Tanganyika, Burundi	OQ407466	OQ407471	present study
***Ergasilus parasarsi* n. sp.**	*Simochromis diagramma*	Cichlidae	Lake Tanganyika, Burundi	OQ407467	OQ407473	present study
*Ergasilus parasiluri*	*Tachysurus fulvidraco*	Bagridae	Dangjiangkou, China	DQ107568	DQ107536	Song *et al*. (2008)
***Ergasilus parvus* n. sp.**	*Spathodus erythrodon*	Cichlidae	Lake Tanganyika, Burundi	OQ407468	OQ407472	present study
*Ergasilus peregrinus*	*Siniperca chuatsi*	Sinipercidae	Dangjiangkou, China	DQ107577	DQ107531	Song *et al*. (2008)
*Ergasilus scalaris*	*Tachysurus dumerili*	Bagridae	Poyang Lake, China	DQ107565	DQ107538	Song *et al*. (2008)
*Ergasilus sieboldi*	*Perca fluviatilis*	Percidae	U Jezu, Czech Republic	MW810238	MW810242	Kvach *et al*. (2021)
*Ergasilus tumidus*	*Acheilognathus taenianalis*	Acheilognathidae	Niushan Lake, China	DQ107569	DQ107535	Song *et al*. (2008)
*Ergasilus wilsoni*	*–*	–	South Korea	KR048765	KR048843	Baek *et al.* (2016)
*Ergasilus yaluzangbus*	*Oxygymnocypris stewartii*	Cyprinidae	Lasa River, Tibet	DQ107578	DQ107540	Song *et al*. (2008)
*Neoergasilus japonicus*	*Lepomis gibbosus*	Centrarchidae	Rohlík, Czech Republic	MH167970	MH167968	Ondračková *et al*. (2019)
*Paraergasilus brevidigitus*	*Cyprinus carpio*	Cyprinidae	Tangxun Lake, China	DQ107576	DQ107530	Song *et al*. (2008)
*Paraergasilus longidigitus*	*Abramis brama*, *Perca fluviatilis*, *Scardinius erythrophthalmus*	Leuciscinae, Percidae,	Pahrbek, U Jezu, Czech Republic	MW810239	MW810243	Kvach *et al*. (2021)
*Paraergasilus medius*	*Ctenopharyngodon idella*	Xenocyprididae	Tangxun Lake, China	DQ107574	DQ107529	Song *et al*. (2008)
*Sinergasilus major*	*Ctenopharyngodon idella*	Xenocyprididae	Tangxun Lake, China	DQ107558	DQ107524	Song *et al*. (2008)
*Sinergasilus polycolpus*	*Hypophthalmichthys molitrix*	Xenocyprididae	Tangxun Lake, China	DQ107563	DQ107525	Song *et al*. (2008)
*Sinergasilus undulatus*	*Cyprinus carpio*	Cyprinidae	Tangxun Lake, China	DQ107561	DQ107526	Song *et al*. (2008)
Lernaeidae						
*Lamproglena chinensis*	*Channa argus*	Channidae	Dangjiangkou, China	DQ107553	DQ107545	Song *et al*. (2008)
*Lamproglena orientalis*	*Chanodichthys dabryi*	Xenocyprididae	Tangxun Lake, China	DQ107549	DQ107542	Song *et al*. (2008)
*Lernaea cyprinacea*	*Chanodichthys erythropterus*	Xenocyprididae	Dongxi Lake, China	DQ107555	DQ107547	Song *et al*. (2008)

Newly generated sequences are given in bold.

## Results

The gill-associated parasitic copepods were obtained from 12 species of cichlids inhabiting Lake Tanganyika. All specimens were assigned to *Ergasilus* on the basis of several diagnostic characters, according to Boxshall and Montú (1997); Boxshall and Halsey (2004); Suárez-Morales and Santana-Piñeros (2008). These included (i) biramous leg IV with 2-segmented exopod and 3-segmented endopod, (ii) 6-segmented antennule, (iii) antenna with a single claw and (iv) the absence of maxillipeds in females. Three new and 2 previously described species of *Ergasilus* are figured and described below.

Species richness of *Ergasilus* parasites on the host species investigated ranged from 1 to 4. *Bathybates ferox* and *Ctenochromis horei* were each found to harbour 1 *Ergasilus* species (i.e. *E. megacheir* and *E. parvus* n. sp., respectively). *Eretmodus marksmithi* and *Lamprologus callipterus* were each found to be hosts of 4 *Ergasilus* species (i.e. *E*. *caparti* n. sp., *E. macrodactylus*, *E*. *parasarsi* n. sp. and *E. parvus* n. sp.). The number of host species ranged from 2 for *E. megacheir* to a maximum of 7 for *E. parvus* n. sp. (Table 3). The highest prevalence (100%) was observed for *E. macrodactylus* on *Gnathochromis permaxillaris* and *Perissodus microlepis*, as well as for *E. parvus* n. sp. on *B. ferox* (only 1 specimen examined). The highest intensity of infection (62 individuals) was observed for *E. caparti* n. sp. on *E. marksmithi* (for detailed information see Table 3).

**Table 3.** Prevalence (first line), mean intensity of infection (second line) and intensity of infection (min–max; third line) of 5 *Ergasilus* species from Lake Tanganyika found on 12 cichlid host species (*n* = total number of host specimens examined)

Parasite species	*B. ferox* (*n* = 1)	*C. horei* (*n* = 11)	*E. marksmithi* (*n* = 28)	*G. permaxillaris* (*n* = 10)	*L. callipterus* (*n* = 15)	*N. brichardi* (*n* = 12)	*N. mondabu* (*n* = 17)	*O. nasuta* (*n* = 3)	*P. microlepis* (*n* = 5)	*S. diagramma* (*n* = 11)	*S. erythrodon* (*n* = 9)	*T. irsacae* (*n* = 7)
*Ergasilus macrodactylus*	–	–	17.8 15.8 (3–38)	100 8.5 (2–16)	60 8.4 (2–15)	–	–	–	100 6.4 (2–13)	–	–	57.1 15.5 (5–28)
*Ergasilus megacheir*	–	9.1 1 (1)	–	–	–	–	–	–	–	36.3 2.5 (2–4)	–	–
*Ergasilus caparti* n. sp.	–	–	50 21.2 (2–62)	–	6.6 1 (1)	8.3 4 (4)	50 3.5 (1–9)	–	–	–	11.1 22 (22)	–
*Ergasilus parasarsi* n. sp.	–	–	3.6 8 (8)	–	6.6 15 (15)	–	–	66.6 6.5 (6–7)	–	9.1 27 (27)	33.3 13.3 (4–24)	42.9 7 (5–9)
*Ergasilus parvus* n. sp.	100 18 (18)	–	32.1 28.5 (11–61)	–	20 11.6 (1–20)	50 1.6 (1–3)	17.6 3 (1–5)	–	–	36.3 5.5 (1–12)	77.7 8.9 (2–19)	–

**Family Ergasilidae (Burmeister, 1835)**

**Genus *Ergasilus* (von Nordmann, 1832)**

***Ergasilus macrodactylus* (Sars, 1909)**

Syn. *Ergasiloides macrodactylus* (Sars, 1909)

***Type-host***: not recorded.

***Type locality***: Lake Tanganyika, Sumbu, Zambia.

***Site on host***: gill filaments.

***Other previous records***: *Brycinus imberi* (Peters, 1852) (Characiformes: Alestidae), *Haplochromis* spp., *Lethrinops* spp., *Pseudotropheus* spp., *Tilapia* spp. (Cichliformes: Cichlidae), Lake Malawi, Malawi (Fryer, 1956).

***Present records***: *E. marksmithi* (localities 1, 2, 4); *G. permaxillaris* (locality 2); *L. callipterus* (localities 2, 4); *P. microlepis* (locality 4); *Tanganicodus irsacae* (locality 2) (see Table 1).

***Comparative material examined***: four voucher specimens of *E. macrodactylus* (Sars, 1909) from *Haplochromis* spp. (Malawi): RBINS COP 6086, 6087, 6088 and 6089.

***Voucher material deposited***: Cr-33 (2 specimens, locality 2); hologenophore: Cr-33 (3 specimens, locality 2).

***Representative DNA sequences***: 18S rDNA (GenBank acc. no. OQ407465) and 28S rDNA (GenBank acc. no. OQ407470) (see also Table 2) sequences from 5 specimens ex *E. marksmithi* (*n* = 1), *G. permaxillaris* (*n* = 3) and *P. microlepis* (*n* = 1).

### Description

**Fig. 2.**
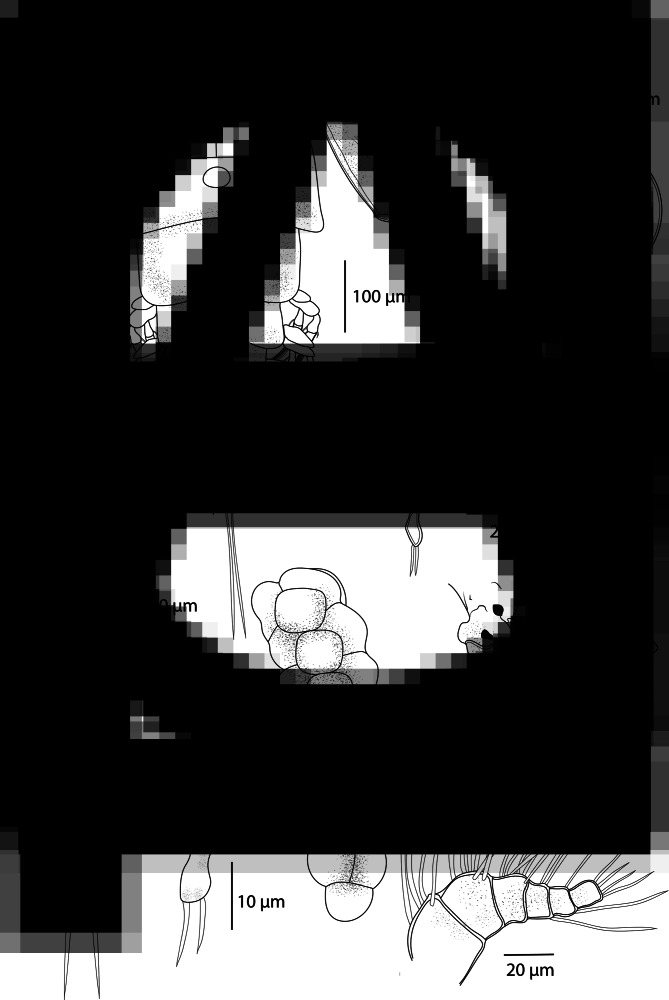
*Ergasilus macrodactylus*, adult female from *Gnathochromis permaxillaris*. (A) Habitus, dorsal; (B) antenna, ventral; (C) mandible and maxilulle, ventral; (D) maxilla, ventral; (E) antennule, ventral; (F) abdomen and caudal rami; (G) egg sac, dorsal; (H) leg V, ventral.

**Fig. 3.**
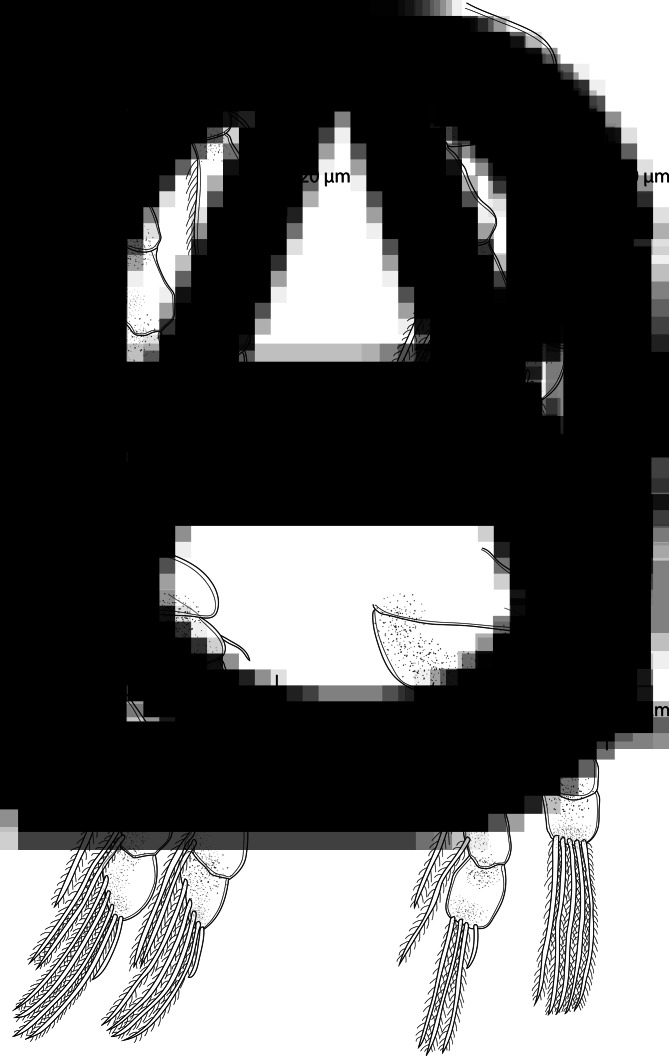
*Ergasilus macrodactylus*, adult female from *Gnathochromis permaxillaris*. (A) Leg I, ventral; (B) leg II, ventral; (C) leg III, ventral; (D) leg IV, ventral.

*Adult female* [based on 10 specimens; Figs 2 and 3]. Body length (measured from anterior margin of prosome to posterior margin of caudal rami) 659 (554–864; *n* = 10). Body comprising prosome and urosome (Fig. 2A). Prosome 5-segmented, composed of cephalosome and 4 pedigerous somites; cephalosome and first pedigerous somite separate.

Cephalosome trapezoidal, with truncate frontal margin and distinctly projecting postero-lateral corners; antennules and antennae visible in dorsal view. Cephalic ornamentation comprising anterior and posterior circular markings of similar sizes; an inverted T-structure of thickened chitin situated postmedially on dorsal side. Eye spot clearly visible near anterior margin of cephalosome. Four pedigerous somites evenly rounded at lateral margins, decreasing in length and width posteriorly.

Urosome narrow, comprising short fifth pedigerous somite, genital segment (Fig. 2F), and 3 free abdominal somites. Genital segment barrel-shaped, posteriorly tapered, with dorsal bilateral cuticular ornamentation. Abdominal somites slightly decreasing in width posteriorly; third abdominal somite slightly incised medially.

Caudal rami with subrectangular shape, slightly longer than wide, posteriorly widening; each ramus armed with 4 terminal setae – the innermost furcal seta at least 5 times longer than others, slightly swollen near the basis and directed outwards. Two egg sacs (Fig. 2G), much longer than wide; each composed of 2–3 rows of circular-shaped eggs and reaching beyond the longest furcal setae.

Antennule (Fig. 2E) 6-segmented; segments well defined and tapering distally, armed with simple setae; setal formula from proximal to distal segments: 4–7–6–3–2–4.

Antenna (Fig. 2B) comprising short coxobasis, 3-segmented endopod and curved terminal claw. First endopod segment twice as long as the coxobasis and attenuated distally; no hyaline border present. Second endopod segment elongated, slightly corrugated in its proximal third. Third endopodal segment small. Terminal claw very slender, pointed and smooth. Antenna without setules or spines.

Mouthparts comprising mandible, maxillule and maxilla; maxilliped absent. Mandible consisting of 3 blades (anterior, middle and posterior); each blade with small teeth on anterior margin (Fig. 2C). Maxillule a small single lobe, with 2 almost equally long distal setae. Maxilla 2-segmented, with numerous sharp teeth on anterior margin of distal segment (Fig. 2D).

Swimming legs I to IV; each comprising coxa, basis and 2 segmented rami (i.e. exopod, endopod) (Fig. 3). Rami of all legs 3-segmented, except 2-segmented exopod of leg IV. Segments distinct, typical of members of the genus. Interpodal plates of all legs lacking spinules. Armature on rami as follows (Roman and Arabic numerals indicating spines and plumose setae, respectively) in Table 4.

**Table 4.** Spine and setal formula of swimming legs of *E. macrodactylus*

	Coxa	Basis	Exopod	Endopod
Leg I	0–0	1–0	I–0; I–1; II–5	0–1; 0–1; II–4
Leg II	0–0	1–0	I–0; 0–1; 0–6	0–1; 0–2; I–4
Leg III	0–0	1–0	0–0; 0–2; I–4	0–1; 0–2; I–4
Leg IV	0–0	1–0	0–0; 0–5	0–1; 0–1; I–3

Leg I (Fig. 3A) coxa unarmed, basis with outer seta. Exopod 3-segmented; first segment with small outer spine; second segment with inner plumose seta and a small outer spine; third segment with small spine on outer corner, longer apical spine and 5 plumose setae. Endopod 3-segmented; first and second segment each with 1 plumose seta; third segment with 4 plumose setae and 2 distal spines.

Leg II (Fig. 3B) coxa unarmed, basis with outer seta. Exopod 3-segmented; first segment with very small outer spine; second segment with 1 plumose seta, lacking spine; third segment with 6 plumose setae. Endopod 3-segmented; first segment with 1 plumose seta; second segment with 2 plumose setae; third segment with 4 plumose setae and 1 distal spine.

Leg III (Fig. 3C) coxa unarmed, basis with outer seta. Exopod 3-segmented; first segment lacking armature; second segment with 2 plumose setae; third segment with 4 plumose setae and 1 distal spine. Endopod 3-segmented; first segment with 1 plumose seta; second segment with 2 plumose setae; third segment with 4 plumose setae and 1 distal spine.

Leg IV (Fig. 3D) coxa unarmed, basis with outer seta. Exopod 2-segmented; first segment longest and without armature; second segment with 5 plumose setae. Endopod 3-segmented; first and second segment each with 1 plumose seta; third segment with 3 plumose setae and 1 distal spine.

Leg V (Fig. 2H) simple and visible, with cylindrical form, bearing 2 terminal simple seta.

Specimens preserved in ethanol rather light in colour; traces of a purple pigment in cephalothorax observed after clearing in lactic acid.

Male: unknown.

### Remarks

*Ergasilus macrodactylus* is characterized, in part, by having trapezoidal cephalothorax, elongate antenna and clearly visible eye spot (Fryer, 1956). Based on the overall body shape, this species is similar to *E. megacheir* but clearly differs from it by having: (i) 3-segmented abdomen; (ii) the first endopod segment of the antenna without hyaline border on anterior edge; (iii) elongate and distally recurved second endopod segment of the antenna (second endopod segment short and twisted in *E. megacheir*); (iv) the terminal claw of the antenna with smooth margins (claw with inner denticle in *E. megacheir*); (v) a tiny spine on the first segment of the exopod of leg II; (vi) 2 plumose setae on the second segment of the endopods of leg II and III (1 plumose seta on the respective endopods in *E. megacheir*); and (vii) a different spine–seta formula on the exopod of leg III (0–0, 0–2, I–4 *vs* 0–0, 0–1, 0–6 in *E. megacheir*).

*Ergasilus macrodactylus* was a cause of much confusion in the past. This species has been described as *Ergasiloides macrodactylus* (Sars, 1909), and later transferred to *Ergasilus* by Fryer (1956), when he found specimens conspecific with *E. macrodactylus* in Lake Malawi. According to Fryer (1956), Sars (1909) was wrong when he based the description of his species on immature specimens, and therefore wrongly placed it in *Ergasiloides*. Although *E. macrodactylus* was already reported in Lake Tanganyika by Sars (1909), it was omitted from the checklist for parasites from Lake Tanganyika (Avenant-Oldewage and Oldewage, 1993). Our findings confirm the presence of *E. macrodactylus* in Lake Tanganyika, even though the overall body size of our specimens is slightly smaller than those of Fryer (1956) (i.e. 659 *vs* 765). In this respect, our specimens of *E. macrodactylus* are more similar to those of Sars (1909).

***Ergasilus megacheir* (Sars, 1909)**

Syn. *Ergasiloides megacheir* (Sars, 1909)

***Type-host***: not recorded.

***Type-locality***: Lake Tanganyika, Sumbu, Zambia.

***Site on host***: gill filaments.

***Other previous records***: *Pterochromis congicus* (Boulenger, 1897) (Cichliformes: Cichlidae), Lake Tumba, Congo System (Fryer, 1964); *Bathybates minor* (Boulenger, 1906), *Bathybates fasciatus* (Boulenger, 1901), *Cyphotilapia frontosa* (Boulenger, 1906), *Haplotaxodon microlepis* (Boulenger, 1906), *Limnotilapia dardenii* (Boulenger, 1899), *Pseudosimochromis curvifrons* (Poll, 1942) (Cichliformes: Cichlidae), *Synodontis multipunctatus* (Boulenger, 1898), *Synodontis granulosus* (Boulenger, 1900) (Siluriformes: Mochokidae), Lake Tanganyika (Fryer, 1965); *Simochromis* sp. (Cichliformes: Cichlidae), Lake Tanganyika (Capart, 1944); unknown hosts, Lake Tanganyika (Sars, 1909; Cunnington, 1920).

***Present records***: ex *C. horei* (locality 4); *Simochromis diagramma* (locality 1) (see Table 1).

***Voucher material deposited***: Cr-34 (1 specimen, locality 1); hologenophore: Cr-34 (1 specimen, locality 1).

***Representative DNA sequences***: 18S rDNA (GenBank acc. no. OQ407466) and 28S rDNA (GenBank acc. no. OQ407471) (see also Table 2) sequences from 2 specimens ex *S. diagramma*.

### Description

**Fig. 4.**
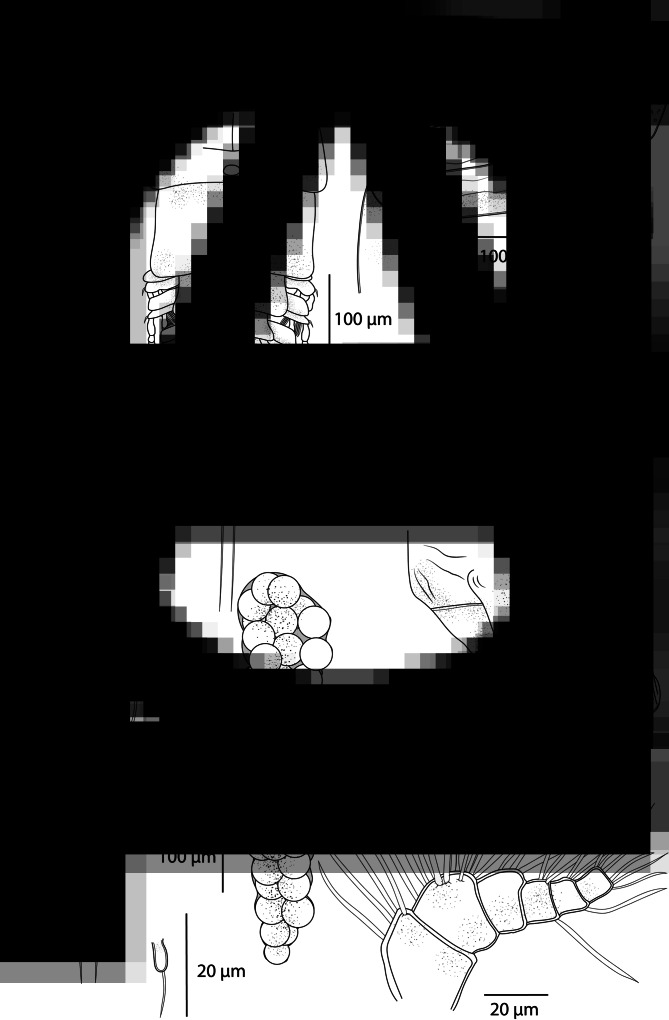
*Ergasilus megacheir*, adult female from *Simochromis diagramma*. (A) Habitus, dorsal; (B) antenna, ventral; (C) mandible and maxilulle, ventral; (D) maxilla, ventral; (E) antennule, ventral; (F) abdomen and caudal rami; (G) egg sac, dorsal; (H) leg V, ventral.

**Fig. 5.**
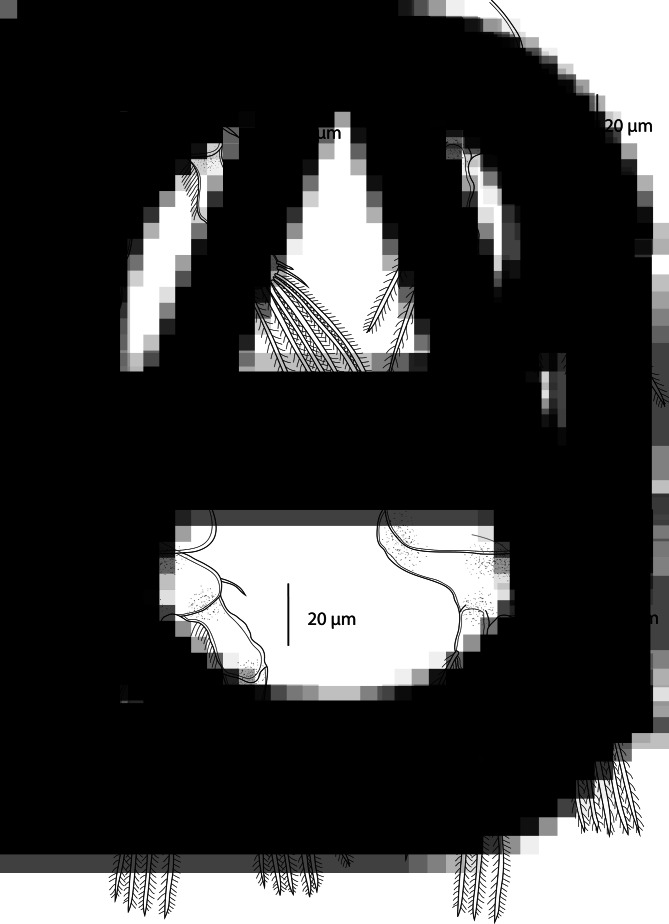
*Ergasilus megacheir*, adult female from *Simochromis diagramma*. (A) Leg I, ventral; (B) leg II, ventral; (C) leg III, ventral; (D) leg IV, ventral.

*Adult female* [based on 5 specimens; Figs 4 and 5]. Body length (measured from anterior margin of prosome to posterior margin of caudal rami) 698 (613–770; *n* = 5). Prosome 5-segmented, composed of cephalosome and 4 pedigerous somites; cephalosome and first pedigerous somite separate (Fig. 4A). Cephalosome quadrangular, frontal edge truncated, postero-lateral corners slightly prominent and rounded; antennules and antennae visible in dorsal view. Cephalic ornamentation comprising anterior ovoid marking and posterior oval marking; an inverted T-structure of thickened chitin situated postmedially on dorsal side, between the circular and oval marking. Eye spot clearly visible near anterior margin of cephalosome. Four pedigerous somites with lateral parts slightly produced backwards, rounded obtusely at the end.

Urosome small, comprising short fifth pedigerous somite, genital segment (Fig. 4F) and 2 free abdominal somites. Fifth pedigerous somite almost wholly concealed. Genital segment barrel-shaped, anteriorly tapered.

Second abdominal somite shallowly incised medially, much longer than preceding somite. Caudal rami subrectangular, as long as the length of the previous segment; each ramus armed with 4 terminal setae – the innermost longest and thickest; outer setae pointing towards middle body axis.

Two egg sacs (Fig. 4G) much longer than wide; each composed of 2–4 rows of circular-shaped eggs. Some specimen egg sacs extremely long, almost the same length as the whole body.

Antennule (Fig. 4E) 6-segmented; segments well defined, tapering distally, armed with simple setae; setal formula from proximal to distal segments: 4–11–3–4–3–6.

Antenna (Fig. 4B) comprising short coxobasis, 3-segmented endopod, and strongly curved terminal claw. First endopodal segment twice as long as the coxobasis, oblong in form, distally slightly narrowed; anterior edge with visible thin hyaline border not fully extending to the base. Second endopodal segment short and twisted, third endopodal segment inconspicuous. Terminal claw about 1/3 length of second endopodal segment, equipped with a recurved denticle inside. Antenna without setules or spines.

Mouthparts comprising mandible, maxillule and maxilla; maxilliped absent. Mandible consisting of 3 blades (anterior, middle and posterior); each blade with small sharp teeth; anterior and posterior blade with teeth located on anterior margin; middle blade with teeth on posterior margin (Fig. 4C). Maxilulle a small lobe with 2 unequally long distal setae and 2 minute inner setae. Maxilla 2-segmented, comprising unarmed syncoxa and basis, distally with numerous sharp teeth on anterior margin (Fig. 4D).

Swimming legs I–IV; each comprising coxa, basis and 2 segmented rami (i.e. exopod, endopod) (Fig. 5). Rami of all legs 3-segmented, except 2-segmented exopod of leg IV. Segments distinct, typical of members of the genus. Interpodal plates of all legs lacking spinules. Armature on rami as follows (Roman and Arabic numerals indicating spines and setae, respectively) in Table 5.

**Table 5.** Spine and setal formula of swimming legs of *E. megacheir*

	Coxa	Basis	Exopod	Endopod
Leg I	0–0	1–0	I–0; I–1; II–5	0–1; 0–1; II–4
Leg II	0–0	1–0	0–0; 0–1; 0–6	0–1; 0–1; I–4
Leg III	0–0	1–0	0–0; 0–1; 0–6	0–1; 0–1; I–4
Leg IV	0–0	1–0	0–0; 0–5	0–1; 0–1; I–3

Leg I (Fig. 5A) coxa unarmed, basis with proximal outer seta. Exopod 3-segmented; first segment with small outer spine; second segment with inner plumose seta and a small outer spine; third segment with small spine on outer corner, longer apical spine and 5 plumose setae. Endopod 3-segmented; first and second segment each with 1 plumose seta; third segment with small spine on outer corner, longer distal spine and 4 plumose setae.

Legs II and III (Fig. 5B and C) similar. Coxa unarmed, basis with proximal outer seta. Exopod with 3 segments; first segment lacking armature; second segment with 1 plumose seta, lacking spine; third segment with 6 plumose setae. Endopod with 3 segments; first and second segment each with 1 plumose seta; third segment with 4 plumose setae and 1 distal spine.

Leg IV (Fig. 5D) coxa unarmed, basis with proximal outer seta. Exopod 2-segmented; first segment longest without armature; second segment with 5 plumose setae. Endopod with 3 segments; first and second segment each with 1 plumose seta; third segment with 3 plumose setae and 1 distal spine.

Leg V (Fig. 4H) extremely small, barely visible, with 1 smooth seta.

Specimens preserved in ethanol brown in colour. No pigment observed after clearing in lactic acid.

Male: unknown.

### Remarks

*Ergasilus megacheir* was already recorded from Lake Tanganyika by Sars (1909), Cunnington (1920), Capart (1944) and Fryer (1965). Fryer (1964) reported this species also from Lake Tumba in Lower Congo. *Ergasilus megacheir* is easily distinguished from other congeners by the presence of a denticle on the inner side of the terminal claw of the antenna. The size of the denticle differs, being more prominent in presumably smaller specimens and less visible in bigger specimens, which supports Fryer's (1965) previous observation. Based on comparative morphology, *E. megacheir* is most similar to *E. macrodactylus*. The differentiation of both species is provided in the remarks for the latter species.

Although *E. megacheir* was the least abundant species in our dataset, its presence in the Lake was reconfirmed after more than 50 year. For the first time, egg sacs were described and drawn. The presence of setae on the basis of legs I–IV was also confirmed. The spine can be seen in the original drawings but did not feature in the spine–seta formula.

***Ergasilus caparti* n. sp.**

***Type-host***: *Neolamprologus brichardi* (Poll, 1974).

***Type-locality***: Magara (3°44′S, 29°19′E), Lake Tanganyika, Burundi.

***Other hosts and localities***: *E. marksmithi* (localities 1, 2), *L. callipterus* (locality 4), *Neolamprologus mondabu* (locality 3), *P. microlepis* (locality 1), *Spathodus erythrodon* (locality 1) (see Table 1).

***Type and voucher material***: holotype (adult female): (Cr-35) ex *N. brichardi*; paratypes (adult females): Cr-35 ex *E. marksmithi*; hologenophores (adult females): Cr-35 ex *S. erythrodon* and *E. marksmithi*.

***Site on host***: gill filaments.

***ZooBank registration***: urn:lsid:zoobank.org:act:7211AE50-81DA-4F73-BE5E-26611C2974C4

***Representative DNA sequences***: 18S rDNA (GenBank acc. no. OQ407469) and 28S rDNA (GenBank acc. no. OQ407474) (see also Table 2) sequences from 5 specimens ex *E. marksmithi* (*n* = 1), *L. callipterus* (*n* = 1), *N. mondabu* (*n* = 1) and *S. erythrodon* (*n* = 2).

***Etymology***: This species is named after André Capart (1917–1993), director of the Royal Institute of Natural Sciences (Belgium), to honour his contributions to knowledge of the crustacean fauna that resulted from expeditions to the African Great Lakes including Lake Tanganyika (1946–1947).

### Description

**Fig. 6.**
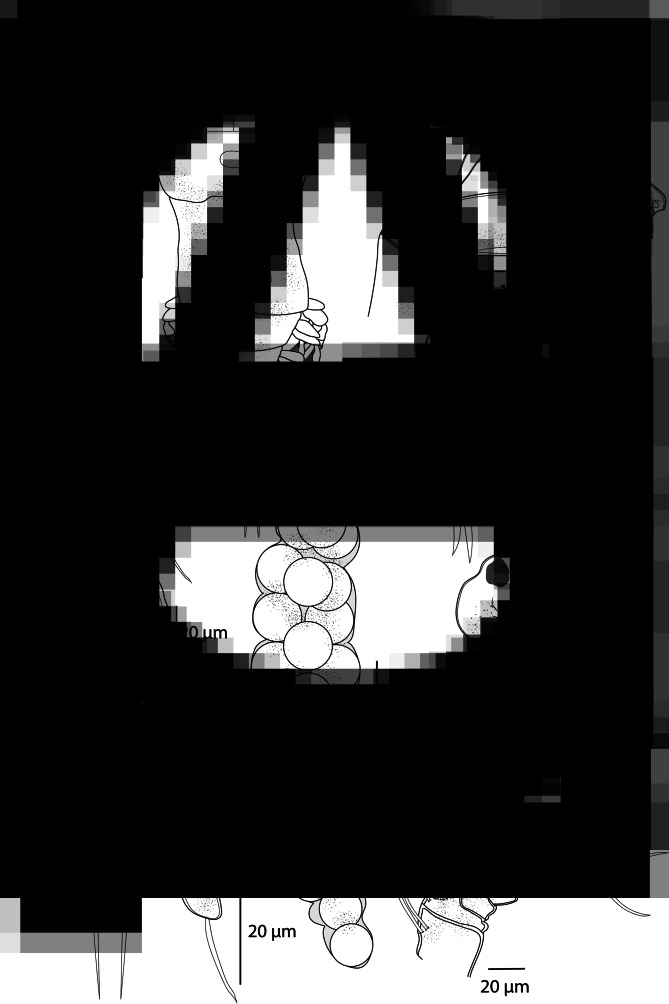
*Ergasilus caparti* n. sp., adult female from *Neolamprologus brichardi*. (A) Habitus, dorsal; (B) antenna, ventral; (C) mandible and maxilulle, ventral; (D) maxilla, ventral; (E) antennule, ventral; (F) abdomen and caudal rami; (G) egg sac, dorsal; (H) leg V, ventral.

**Fig. 7.**
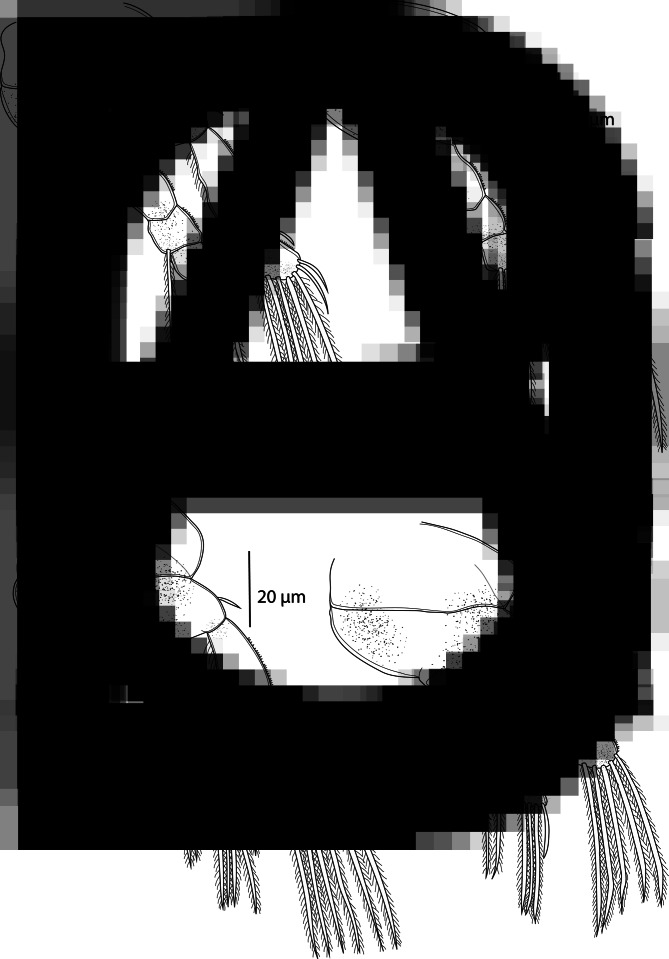
*Ergasilus caparti* n. sp., adult female from *Neolamprologus brichardi*. (A) Leg I, ventral; (B) leg II, ventral; (C) leg III, ventral; (D) leg IV, ventral.

*Adult female* [based on 10 specimens; Figs 6 and 7]. Body length (measured from anterior margin of prosome to posterior margin of caudal rami) 509 (411–611; *n* = 10). Prosome 5-segmented, composed of cephalosome and 4 pedigerous somites; cephalosome and first pedigerous somite separate. Cephalosome hexagonal, with a medial indentation on posterior margin; antennules and antennae visible in dorsal view (Fig. 6A). Cephalic ornamentation comprising anterior circular marking and a more posterior, slightly larger oval marking; an inverted T-structure of thickened chitin situated medially on dorsal side, between the circular and oval marking. First pedigerous somite of similar size as cephalosome, slightly tapering medially. Second to fourth pedigerous somites each markedly narrowing posteriorly.

Urosome comprising short fifth pedigerous somite, genital segment (Fig. 6F), and 2 free abdominal somites. Genital segment of similar shape to the first pedigerous somite, slightly widening in posterior half, ventral surface ornate with spinules along postero-ventral margin. Two free abdominal somites with a row of acute spinules along posterior margin on ventral surface. First abdominal somite short, slightly longer than the fifth pedigerous somite. Second abdominal somite deeply incised medially, slightly larger than the preceding somite.

Caudal rami subrectangular, slightly longer than wide; each ramus bearing 4 terminal setae – the innermost longest and thickest but not exceeding egg sacs. Two egg sacs (Fig. 6G) quite long and narrowing distally; each composed of 2–4 rows of eggs.

Antennule (Fig. 6E) 6-segmented, tapering distally, armed with simple setae; setal formula from proximal to distal segments: 2–10–3–3–2–4.

Antenna (Fig. 6B) comprising short coxobasis, 3-segmented endopod and strongly curved terminal claw. First endopodal segment longest; second endopodal segment proximally with conspicuous indentation of cuticle on outer side, formed by 2 ridges crossing each other. Third endopodal segment small, but more conspicuous under the light microscope than in other species from Lake Tanganyika. Terminal claw about 1/3 length of the second endopodal segment. Antenna without setules or spines.

Mouthparts (Fig. 6C and D) comprising mandible, maxilulle and maxilla; maxilliped absent. Mandible consisting of 3 blades (anterior, middle and posterior); anterior blade with sharp teeth on anterior margin; middle and posterior blade with teeth along posterior margin. Maxilulle a well-developed lobe, bearing 2 almost equally long smooth setae. Maxilla 2-segmented, comprising unarmed syncoxa and basis, distally with numerous sharp teeth on anterior margin.

Swimming legs I–IV; each comprising coxa, basis and 2 segmented rami (i.e. exopod, endopod) (Fig. 7). Rami of all legs 3-segmented, except 2-segmented exopod of leg IV. Segments distinct, typical of members of the genus. Interpodal plates of all legs lacking spinules. Armature on rami as follows (Roman and Arabic numerals indicating spines and setae, respectively) in Table 6.

**Table 6.** Spine and setal formula of swimming legs of *E. caparti* n. sp.

	Coxa	Basis	Exopod	Endopod
Leg I	0–0	1–0	0–0; I–1; II–5	0–0; 0–1; II–4
Leg II	0–0	1–0	0–0; 0–1; 0–6	0–1; 0–1; I–4
Leg III	0–0	1–0	0–0; 0–1; 0–6	0–1; 0–1; I–4
Leg IV	0–0	1–0	0–0; 0–5	0–1; 0–1; I–3

Leg I (Fig. 7A) coxa unarmed, basis with proximal outer seta. Exopod 3-segmented; first segment lacking armature; second segment with inner plumose seta and a small outer spine; third segment with small spine on outer corner, longer apical spine and 5 plumose setae. Endopod 3-segmented; first segment lacking armature; second segment with 1 plumose seta; third segment with 4 plumose setae and 2 distal spines. Outer margins of both rami partly or completely covered with rows of spinules.

Legs II and III similar (Fig. 7B and C). Coxa unarmed, basis with proximal outer seta. Exopod 3-segmented; first segment lacking armature; second segment with 1 plumose seta, lacking spine; third segment with 6 plumose setae. Endopod 3-segmented; first and second segment each with 1 plumose seta; third segment with 4 plumose setae and 1 distal spine. Outer margins of both rami partly or completely covered with rows of spinules.

Leg IV (Fig. 7D) coxa unarmed, basis with proximal outer seta. Exopod 2-segmented; first segment longest without armature; second segment with 5 plumose setae. Endopod 3-segmented; first and second segment each with 1 plumose seta; third segment with 3 plumose setae and 1 distal spine. Outer margins of both rami partly or completely covered with rows of spinules.

Leg V (Fig. 6H) reduced but visible, bearing 1 simple seta.

Specimens preserved in ethanol faint brown in colour, sometimes with dark brown spots in the cephalothorax.

Male: unknown.

### Remarks

*Ergasilus caparti* n. sp. shows greatest similarity to *E. cunningtoni* (Capart, 1944), a widely distributed copepod reported from fishes of many families, namely Alestidae, Cichlidae, Clupeidae, Cyprinidae, Distichodontidae, Mochokidae, Mormyridae (*Campylomormyrus elephas* – type host species) and Schilbeidae, from the Congo River System (Capart, 1944; Fryer, 1964, 1967), the Galma River (Shotter, 1977) and Lake Volta (Paperna, 1969). In both species, the second endopodal segment of antenna has a conspicuous indentation of cuticle on outer side, formed by 2 ridges crossing each other. *Ergasilus caparti* n. sp. is differentiated from *E. cunningtoni* by: (i) having a much smaller body size (509 *vs* 970); (ii) having 2-segmented abdomen (*vs* 3-segmented abdomen in *E. cunningtoni*); (iii) the presence of a spine on the basis of legs III and IV; (iv) the absence of a spine on the first segment of the exopod of leg I; (v) the absence of seta on the first segment of endopod of leg I; (vi) the absence of a spine on the first segment of exopods of legs II and III; (vii) having only 1 seta on the second segments of the endopods of legs II, III and IV (*vs* 2 setae in *E. cunningtoni*); (viii) having a less prominent eyespot; (ix) the presence of spinules on the outer margins of both rami of all legs (*vs* both rami of all legs with smooth margins in *E. cunningtoni*).

***Ergasilus parasarsi* n. sp.**

***Type-host***: *S. diagramma* (Günther, 1894)

***Type-locality***: Magara (3°44′S, 29°19′E), Lake Tanganyika, Burundi.

***Other hosts and localities***: *E. marksmithi* (locality 1), *G. permaxillaris* (locality 2), *L. callipterus* (locality 2), *Ophthalmotilapia nasuta* (locality 1), *P. microlepis* (locality 4), *T. irsacae* (locality 2) (see Table 1).

***Type and voucher material***: holotype (adult female): (Cr-36) ex *S. diagramma*; paratypes (adult females): Cr-36 ex *T. irsacae*; hologenophores (adult females): Cr-36 ex *O. nasuta* and *T. irsacae*.

***Site on host***: gill filaments.

***Zoobank registration***: urn:lsid:zoobank.org:act:E915E962-E162-4331-BB72-4CE17ADFE5E5

***Representative DNA sequences***: 18S rDNA (GenBank acc. no. OQ407467) and 28S rDNA (GenBank acc. no. OQ407473) (see also Table 2) sequences from 4 specimens ex *L. callipterus* (*n* = 2), *O. nasuta* (*n* = 1) and *T. irsacae* (*n* = 1).

***Etymology***: The specific name reflects the close morphological resemblance of the new species to *E. sarsi* (Capart, 1944).

### Description

**Fig. 8.**
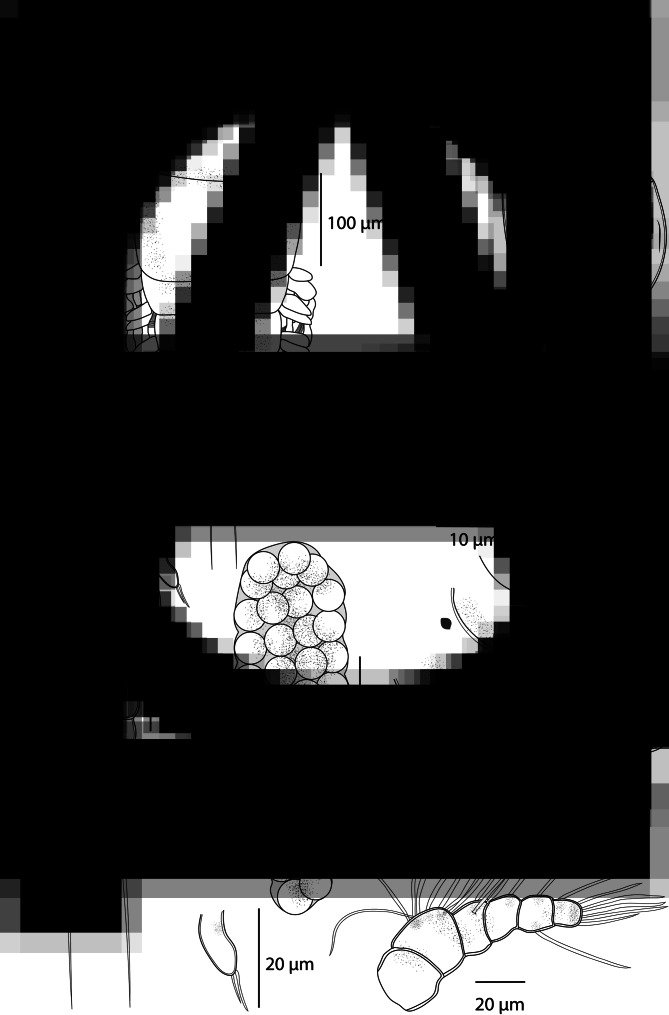
*Ergasilus parasarsi* n. sp., adult female from *Simochromis diagramma*. (A) Habitus, dorsal; (B) antenna, ventral; (C) mandible and maxilulle, ventral; (D) maxilla, ventral; (E) antennule, ventral; (F) abdomen and caudal rami; (G) egg sac, dorsal; (H) leg V, ventral.

**Fig. 9.**
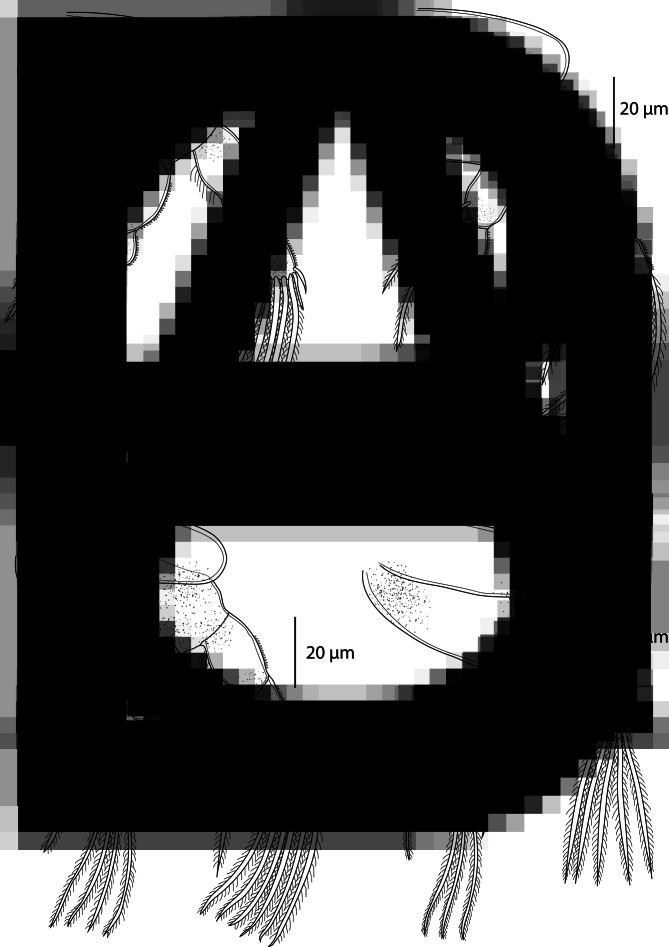
*Ergasilus parasarsi* n. sp., adult female from *Simochromis diagramma*. (A) Leg I, ventral; (B) leg II, ventral; (C) leg III, ventral; (D) leg IV, ventral.

*Adult female* [based on 10 specimens; Figs 8 and 9]. Body length (measured from anterior margin of prosome to posterior margin of caudal rami) 575 (455–694; *n* = 10). Body comprising prosome and urosome (Fig. 8A). Prosome 5-segmented, consisting of cephalosome and 4 pedigerous somites; cephalosome and first pedigerous somite separate.

Cephalosome slightly longer than wide, oval to trapezoidal, with antennules and antennae visible in dorsal view. Cephalic ornamentation comprising anterior circular marking and an inverted T-structure of thickened chitin situated medially on dorsal side. First pedigerous somite almost the same length as cephalosome; the other 3 pedigerous somites markedly shorter than the first one, decreasing in length and width posteriorly. Urosome comprising short fifth pedigerous somite, barrel-shaped genital segment (Fig. 8F) and 2 free abdominal somites. Genital segment with dorsal bilateral marking in the form of a stripe running lengthwise and a more laterally situated elongate spot, ventral surface ornate with spinules along postero-ventral margin. Two free abdominal somites with a row of acute spinules along posterior margin on ventral surface. First abdominal somite of similar length as the fifth pedigerous somite. Second abdominal somite deeply incised medially, much larger than the preceding somite. Caudal rami subrectangular, slightly wider than long; each ramus bearing 4 terminal setae – the innermost longest and thickest. Two cylindrical egg sacs (Fig. 8G) relatively short, not exceeding the longest furcal seta.

Antennule (Fig. 8E) 6-segmented, tapering distally, armed with simple setae; setal formula from proximal to distal segments: 0–8–3–1–2–6.

Antenna (Fig. 8B) prehensile, comprising short coxobasis, 3-segmented endopod and strongly curved terminal claw. First endopodal segment longest; second endopodal segment proximally with indentation of cuticle on outer side; third endopodal segment inconspicuous. Terminal claw about 1/4 length of second endopodal segment. Antenna without setules or spines.

Mouthparts (Fig. 8C and D) comprising mandible, maxillule and maxilla; maxilliped absent. Mandible consisting of 3 blades (anterior, middle and posterior); each blade with sharp teeth on anterior margin. Maxillule a single small lobe, bearing 2 almost equally long smooth setae. Maxilla 2-segmented; syncoxa unarmed; basis relatively slender, distally possessing numerous sharp teeth on anterior margin.

Swimming legs I to IV; each comprising coxa, basis and 2 segmented rami (i.e. exopod, endopod) (Fig. 9). Rami of all legs 3-segmented, except 2-segmented exopod of leg IV. Segments distinct, typical of members of the genus. Interpodal plates of all legs lacking spinules. Armature on rami as follows (Roman and Arabic numerals indicating spines and setae, respectively) in Table 7.

**Table 7.** Spine and setal formula of swimming legs of *E. parasarsi* n. sp.

	Coxa	Basis	Exopod	Endopod
Leg I	0–0	0–0	I–0; 0–1; II–5	0–1; 0–1; II–4
Leg II	0–0	0–0	0–0; 0–1; 0–6	0–1; 0–1; I–4
Leg III	0–0	0–0	0–0; 0–1; 0–6	0–1; 0–1; I–4
Leg IV	0–0	0–0	0–1; 0–5	0–1; 0–1; I–3

Leg I (Fig. 9A) coxa and basis unarmed. Exopod 3-segmented; first segment with small outer spine; second segment with inner plumose seta, lacking spine; third segment with small spine on outer corner, longer apical spine and 5 plumose setae. Endopod 3-segmented; first segment and second segment each with 1 plumose seta, lacking spine; third segment with 4 plumose setae and 2 distal spines. Outer margins of both rami partly or completely covered with rows of spinules.

Legs II and III similar (Fig. 9B and C). Coxa and basis unarmed. Exopod 3-segmented; first segment lacking armature; second segment with 1 plumose seta, lacking spine; third segment with 6 plumose setae. Endopod 3-segmented; first and second segment each with 1 plumose seta; third segment with 4 plumose setae and 1 distal spine. Outer margins of both rami partly or completely covered with rows of spinules.

Leg IV (Fig. 9D) coxa and basis unarmed. Exopod 2-segmented; first segment longest without armature; second segment with 5 plumose setae. Endopod 3-segmented; first and second segment each with 1 plumose seta; third segment with 3 plumose setae and 1 distal spine. Outer margins of both rami partly or completely covered with rows of spinules.

Leg V (Fig. 8H) reduced but visible, bearing 2 simple setae located distally near each other.

Specimens preserved in ethanol rather light in colour. Traces of blue pigment in cephalothorax (i.e. cephalosome plus first pedigerous somite) observed after clearing in lactic acid.

Male: unknown.

### Remarks

*Ergasilus parasarsi* n. sp. resembles *E. sarsi* that was described from the cichlid *Tylochromis mylodon* (Regan, 1920) from Lake Mweru, Congo Basin, DR Congo (Capart, 1944; Fryer, 1968), and also reported from Lake Tanganyika without mentioning of host species (Sars, 1909; Cunnington, 1920; Capart, 1944). It has also been found in Lake Bangwelu (Fryer, 1959), the Volta Basin (Paperna, 1969) and the River Galma in Niger (Shotter, 1977) from fishes of 5 families, namely Cichlidae, Clariidae, Mochokidae, Mormyridae and Poeciliidae. Detailed morphological comparison of our specimens with the type specimens of *E. sarsi* showed their non-conspecificity. *Ergasilus parasarsi* n. sp. differs from *E. sarsi* by the following characters: (i) different proportions of the first and second endopodal segments of the antenna (first segment much longer than second in *E. sarsi*); (ii) presence of a spine on the first segment of the exopod of leg I; (iii) the absence of a spine on the first segment of the exopod of leg IV; (iv) having only 1 seta on the second segment of the exopods of legs II, III and IV (*vs* 2 setae in *E. sarsi*); and (v) the presence of spinules on the outer margins of both rami of all legs (*vs* both rami of all legs with smooth margins in *E. sarsi*).

***Ergasilus parvus* n. sp.**

***Type-host***: *S. erythrodon* (Boulenger, 1900)

***Type-locality*:** Magara (3°44′S, 29°19′E), Lake Tanganyika, Burundi.

***Other hosts and localities***: *B. ferox* (fish market in Burundi), *E. marksmithi* (localities 1, 4), *L. callipterus* (locality 1), *N. brichardi* (locality 1), *N. mondabu* (locality 4) (see Table 1).

***Type and voucher material***: holotype (adult female): (Cr-37) ex *S. erythrodon*; paratype (adult female): (Cr-37) ex *S. erythrodon*; hologenophores (adult females): Cr-37 ex. *S. erythrodon* and *L. callipterus*.

***Site on host***: gill filaments.

***Zoobank registration***: urn:lsid:zoobank.org:act:40C5AD22-CF1E-4873-8F58-E0774928E368

***Representative DNA sequences***: 18S rDNA (GenBank acc. no. OQ407468) and 28S rDNA (GenBank acc. no. OQ407472) (see also Table 2) sequences from 6 specimens ex *B. ferox* (*n* = 1), *E. marksmithi* (*n* = 2), *N. brichardi* (*n* = 1) and *S. erythrodon* (*n* = 2).

***Etymology***: The specific name (an adjective) is from Latin (*parvus* = small) and refers to the body size.

### Description

**Fig. 10.**
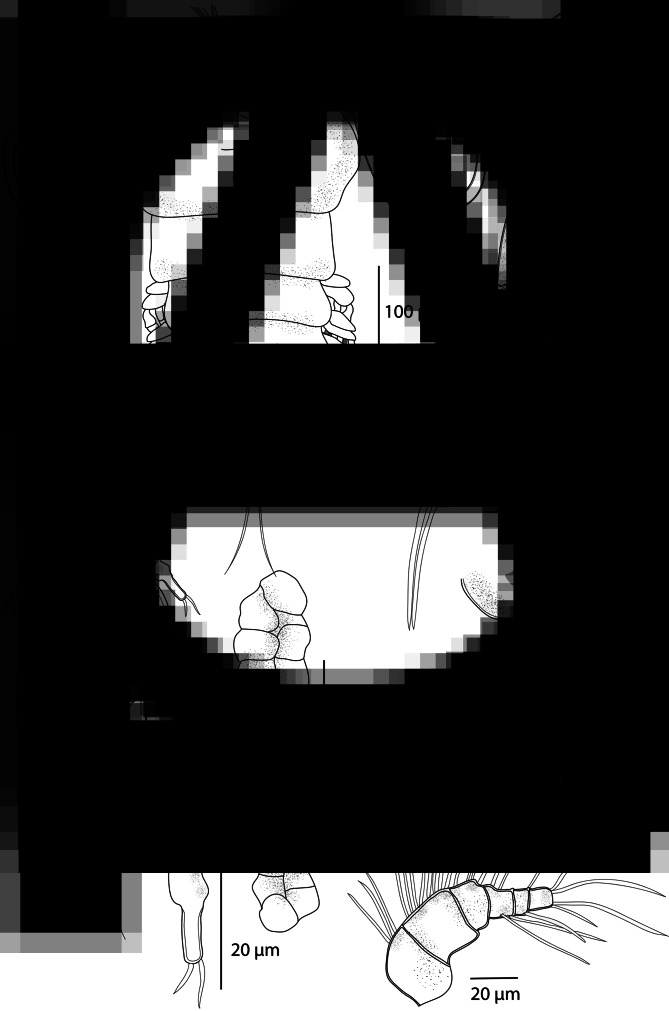
*Ergasilus parvus* n. sp., adult female from *Spathodus erythrodon*. (A) Habitus, dorsal; (B) antenna, ventral; (C) mandible and maxilulle, ventral; (D) maxilla, ventral; (E) antennule, ventral; (F) abdomen and caudal rami; (G) egg sac, dorsal; (H) leg V, ventral.

**Fig. 11.**
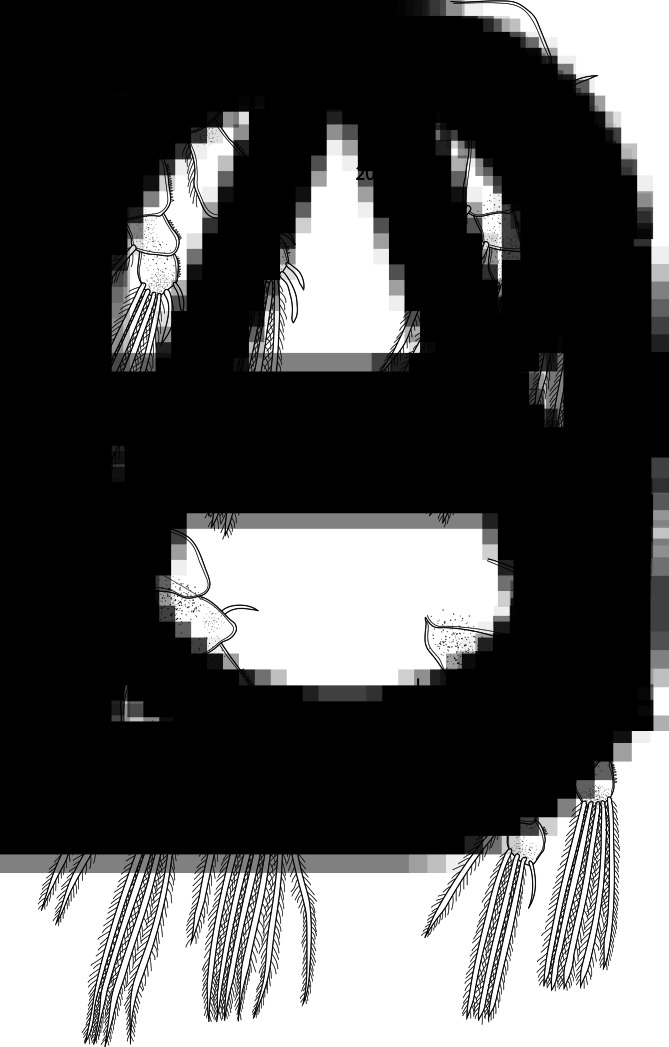
*Ergasilus parvus* n. sp., adult female from *Spathodus erythrodon*. (A) Leg I, ventral; (B) leg II, ventral; (C) leg III, ventral; (D) leg IV, ventral.

*Adult female* [based on 10 specimens; Figs 10 and 11]. Body length (measured from anterior margin of prosome to posterior margin of caudal rami) 475 (417–533; *n* = 10). Body comprising prosome and urosome (Fig. 10A). Prosome 5-segmented, composed of cephalosome and 4 pedigerous somites; cephalosome and first pedigerous somite separate. Cephalosome slightly wider than long, bluntly rounded anteriorly, bulged laterally, indented in posterior third, with antennules and antennae visible in dorsal view. Cephalic ornamentation comprising anterior circular marking and a more posterior, less visible oval marking; an inverted T-structure of thickened chitin situated medially on dorsal side, between the circular and oval marking. First 4 pedigerous somites well developed, decreasing in length and width posteriorly. Urosome comprising short fifth pedigerous somite, narrowed genital segment in posterior third (Fig. 10F) and 2 free abdominal somites decreasing in width posteriorly. Second abdominal somite incised medially, about as long as preceding somite. Caudal rami subrectangular, slightly longer than wide; each ramus armed with 3 terminal setae – the innermost longest and thickest. Two long egg sacs (Fig. 10G), much longer than wide, reaches past longest furcal seta; each composed of 2 rows of brick-shaped eggs.

Antennule (Fig. 10E) 6-segmented, tapering distally, armed with simple setae; setal formula from proximal to distal segments: 3–8–4–4–2–4.

Antenna (Fig. 10B) prehensile, composed of short coxobasis, 3-segmented endopod and strongly curved terminal claw. First endopodal segment markedly more robust than the second one; second endopodal segment and terminal claw evenly curved ventrally; third endopodal segment inconspicuous. Terminal claw about 1/5 length of second endopodal segment. Antenna without setules, spines or indentations.

Mouthparts (Fig. 10C and D) comprising mandible, maxillule and maxilla; maxilliped absent. Mandible consisting of 3 blades (anterior, middle and posterior); each blade with sharp teeth on anterior margin. Maxilulle a single lobe, bearing 2 equally long setae. Maxilla 2-segmented, comprising unarmed syncoxa and basis; basis distally with numerous sharp teeth on anterior margin.

Swimming legs I–IV; each comprising coxa, basis and 2 segmented rami (i.e. exopod, endopod) (Fig. 11). Rami of all legs 3-segmented, except 2-segmented exopod of leg IV. Segments distinct, typical of members of the genus. Interpodal plates of all legs lacking spinules. Armature on rami as follows (Roman and Arabic numerals indicating spines and setae, respectively) in Table 8.

**Table 8.** Spine and setal formula of swimming legs of *E. parvus* n. sp.

	Coxa	Basis	Exopod	Endopod
Leg I	0–0	1–0	I–0; I–1; II–5	0–1; 0–1; II–4
Leg II	0–0	1–0	0–0; 0–1; 0–6	0–1; 0–1; I–4
Leg III	0–0	1–0	0–0; 0–1; 0–6	0–1; 0–1; I–4
Leg IV	0–0	1–0	0–0; 0–5	0–0; 0–1; I–3

Leg I (Fig. 11A) with unarmed coxa and basis with outer seta. Exopod 3-segmented; first segment with small outer spine; second segment with inner plumose seta and small outer spine; third segment with small spine on outer corner, long apical spine and 5 plumose setae. Endopod 3-segmented; first and second segment each with plumose inner seta; third segment with small spine on outer corner, long apical spine and 4 plumose setae. Outer margins of both rami partly or completely covered with rows of spinules.

Legs II and III similar (Fig. 11B and C). Coxa unarmed, basis with outer seta. Exopod 3-segmented; first segment lacking armature; second segment with 1 plumose seta, lacking spine; third segment with 6 setae. Endopod 3-segmented; first and second segment each with 1 plumose seta; third segment with 4 plumose setae and 1 distal spine. Outer margins of both rami partly or completely covered with rows of spinules.

Leg IV (Fig. 11D) with unarmed coxa and basis having outer seta. Exopod 2-segmented; first segment longest with no armature; second segment with 5 plumose setae. Endopod 3-segmented; first segment lacking armature; second segment with 1 plumose seta; third segment with 3 plumose setae and distal spine. Outer margins of both rami partly or completely covered with rows of spinules.

Leg V (Fig. 10H) reduced but visible, bearing 2 simple seta located distally.

Specimens preserved in ethanol faint brown in colour; traces of a purple pigment in cephalosome sometimes observed after clearing in lactic acid.

Male: unknown.

### Remarks

Based on the body shape and antenna morphology, *E. parvus* n. sp. is most similar to *E. latus* (Fryer, 1960), described from the mouthbrooding cichlid *Oreochromis niloticus* (Linnaeus, 1758) from Lake Turkana, Kenya (Fryer, 1960). Records of the latter species came also from Ghana (Thurston, 1970), the Niki River (Fryer, 1968), the River Galma (Shotter, 1977), Kitona (Fryer, 1963) and the Afram Basin, Mawli River and Peshi Lagoon (Paperna, 1969). *Ergasilus parvus* n. sp. is differentiated from *E. latus* by having: (i) a much smaller body size (475 *vs* 900); (ii) a small outer spine on the exopod of leg I; (iii) a seta on the second endopod segment of leg II; (iv) brick-shaped egg sacs (*vs* ovoid egg sacs in *E. latus*); (v) a less prominent leg V; and (vi) the presence of cephalic ornamentation (ovoid and circular marking) (*vs* only inverted T-structure present in *E. latus*).

### Molecular characterization and phylogenetic position of *Ergasilus* species from Lake Tanganyika within the Ergasilidae

No intraspecific variability was detected for partial 18S and 28S rDNA sequences of *Ergasilus* species parasitizing cichlid fishes from Lake Tanganyika. The overall *p*-distance among *Ergasilus* species found for 18S and 28S rDNA sequences was 0.1 and 2% (see Table 9), respectively. Moreover, the 18S sequences displayed no or low (1 bp) interspecific variability; no differences were found among 18S rDNA sequences of *E. macrodactylus*, *E. parvus* n. sp. or *E*. *caparti* n. sp., or between sequences of *E. megacheir* or *E*. *parasarsi* n. sp. *Ergasilus parasarsi* n. sp. was revealed as the most genetically distant species to *E. caparti* n. sp. or *E. megacheir* (2.72%). The lowest values of interspecific differences (0.85%) were found between *E. macrodactylus* and *E. parvus* n. sp. or *E. megacheir*.

**Table 9.** Nucleotide comparison of the partial 28S rDNA sequences of family Ergasilidae based on 589 bp-long alignment

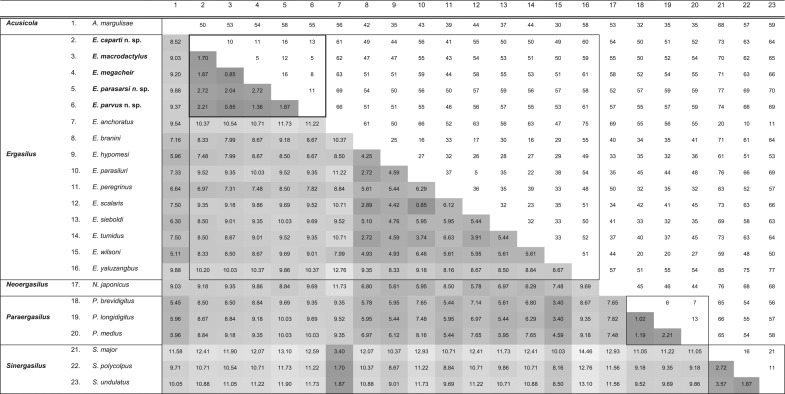																							

*P*-distance (%) is given below the diagonal and the number of variable nucleotides above the diagonal. Conditional formatting highlights the lowest *p*-distances in dark grey while higher values in light grey.

**Fig. 12.**
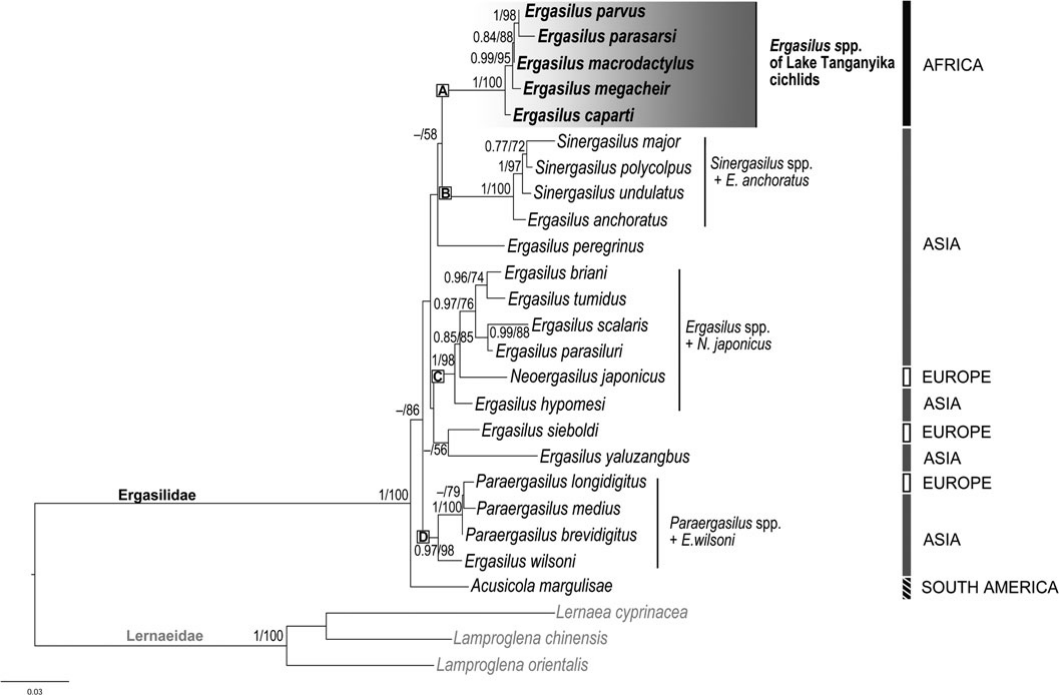
Phylogenetic tree of Ergasilidae reconstructed by maximum likelihood. The tree is based on the combined sequences of partial genes coding 18S and 28S rRNA. Values along the branches indicate posterior probabilities from Bayesian inference and bootstrap values from maximum likelihood (dashes indicate values below 0.7 and 50, respectively). Letters (A)–(D) represent well-supported group of Ergasilidae.

ML and BI analyses based on concatenated alignment including partial 18S and 28S rDNA sequences of Ergasilidae yielded trees with congruent topologies with similar nodal support values and revealed 4 well-supported groups (Fig. 12): (A) *Ergasilus* species from Lake Tanganyika cichlids, (B) *Sinergasilus* species and the *E. anchoratus* group, (C) Asian *Ergasilus* species and the *Neoergasilus japonicus* group and (D) *Paraergasilus* species and the *E. wilsoni* group. The present results are consistent with previously reported ergasilid phylogenies (Song *et al*., 2008; Santacruz *et al*., 2020; Kvach *et al*., 2021) and suggest the polyphyletic status of the genus *Ergasilus*. Although *Ergasilus* species from Lake Tanganyika cichlids formed a well-supported clade, their position within Ergasilidae was not fully resolved due to the observed low support values as well as an insufficient amount of molecular data.

#### Key for identification of *Ergasilus* species from Lake Tanganyika

(2) Five-segmented antennule3
(1) Six-segmented antennule5
(4) Second endopodal segment of antenna with outer denticle near its distal end; leg V 2-segmented, with 1 seta on reduced proximal segment and 2 setae on distal segment; caudal ramus with 1 long and 3 short setae.*E. flaccidus*
(3) Second endopodal segment of antenna with inner denticle near its proximal end; leg V reduced to 1 segment, with 4 setae; caudal ramus with 2 long and 2–3 short setae*E. kandti*
(6) Each caudal ramus with 3 setae7
(5) Each caudal ramus with 4 setae9
(8) Second endopodal segment of antenna of similar diameter as first endopodal segment, with slight outer depression near its proximal end; cephalosome with smooth lateral margins, inverted T-structure having anteriorly recurved lateral ends (resembling a smile); gap between cephalosome and first thoracic segment clearly marked; leg V with 3 setae; 3-segmented abdomen*E. sarsi*
(7) Second endopodal segment of antenna much thinner than first endopodal segment, without depression; cephalosome bulged laterally, with inverted T-structure lacking anteriorly recurved lateral ends; leg V with 2 setae; 2-segmented abdomen*E. parvus*
(10) Second endopodal segment of antenna without depression; cephalosome trapezoidal, with inverted T-structure between circular and ovoid marking; leg V cylindrical, with 2 setae; 3-segmented abdomen; caudal ramus with 1 long and 3 short setae*E. macrodactylus*
(9) Second endopodal segment of antenna with outer proximal depression11
(12) Terminal claw of antenna with small inner denticle; second endopodal segment of antenna short and twisted; anterior edge of first endopodal segment of antenna with thin hyaline border; cephalosome quadrangular, with inverted T-structure between circular and ovoid marking; leg V extremely small, with 1 seta; 2-segmented abdomen*E. megacheir*
(11) Terminal claw of antenna without a denticle13
(14) Second endopodal segment of antenna with conspicuous depression formed by 2 ridges crossing each other; cephalosome hexagonal, posterior margin with medial indentation, with inverted T-structure between circular and ovoid marking; first pedigerous somite slightly tapering medially, with inverted U marking; leg V with 1 seta*E. caparti*
(13) Second endopodal segment of antenna with indentation; cephalosome trapezoidal, with circular marking anterior to inverted T-structure; first pedigerous somite slightly tapering posteriorly; leg V with 2 setae*E. parasarsi*

## Discussion

Our investigation of crustacean parasites of cichlid fishes from Lake Tanganyika revealed the presence of 2 previously described and 3 new species of *Ergasilus* that were identified using a combined morphological and molecular approach. The occurrence of both previously described species (*E. macrodactylus* and *E. megacheir*) had already been confirmed in Lake Tanganyika in previous studies (Sars, 1909; Cunnington, 1920; Capart, 1944; Fryer, 1965); host species for *E. macrodactylus* were not recorded (Sars, 1909), and *E. megacheir* was found on 6 cichlids and 2 mochochid species of the genus *Synodontis* (Sars, 1909; Cunnington, 1920; Capart, 1944; Fryer, 1965). The present study brings new host records for these species: *E. marksmithi*, *G. permaxillaris*, *L. callipterus*, *P. microlepis* and *T. irsacae* for *E. macrodactylus* and *C. horei* and *S. diagramma* for *E. megacheir*. The occurrence of both these species has also been recorded in other areas of Africa; *E. megacheir* was collected from the gills of 1 cichlid species, *P. congicus* (Boulenger, 1897), in Lake Tumba in the Lower Congo (Fryer, 1964) and the presence of *E. macrodactylus* was confirmed in Lake Malawi on 4 cichlid species and 1 alestid (Fryer, 1956). However, the first description of *E. macrodactylus* as well as its occurrence in Lake Tanganyika (Sars, 1909) is doubtful, similarly to the description of *E. brevimanus* (Sars, 1909). The present results confirmed the presence of *E. macrodactylus* in Lake Tanganyika for the first time since Sars (1909), even though the overall size of the body is smaller than Fryer's redescription (Fryer, 1956) from Lake Malawi. The main difference between both descriptions is the segmentation of the antennule (Sars mentions 5-segmented antennule, Fryer 6-segmented antennule). From this point of view, the material from Lake Tanganyika matches the Fryer's description, since the antennule is clearly 6-segmented.

Until now, there were no studies on the genetic characteristics of African *Ergasilus* species; thus, the molecular data presented here represent the first insight into the phylogenetic relationships of this genus in Africa. In contrast to data on the well-documented distribution of ergasilid copepods among host species and regions, molecular data for this large taxon remain scarce over the whole range of its global distribution. Among the 30 valid Ergasilidae genera, molecular data are currently only available for 7 [*Acusicola* (Cressey, 1970); *Ergasilus*; *Gamispinus* (Thatcher and Boeger, 1984); *Miracetyma* (Malta, 1993); *Neoergasilus* (Yin, 1956); *Paraergasilus* (Markevich, 1937); *Sinergasilus* (Yin, 1949) and *Therodamas* (Krøyer, 1863)]. Moreover, only molecular data belonging to species with an Asian origin were available for a long time (Song *et al*., 2008). Recently, molecular data for the cosmopolitan species *E. sieboldi* (von Nordmann, 1832) collected from European perch in the Czech Republic, and *E. yandemontei* (Waicheim, Mendes Marques, Rauque and Viozzi, 2021) from atherinid silversides in Argentina and for a few other genera of Ergasilidae have been published (Ondračková *et al*., 2019; Santacruz *et al*., 2020; Kvach *et al*., 2021; Waicheim *et al*., 2021).

Based on the possibility to compare the genetic data of representatives of Ergasilidae, which are available in the GenBank database, molecular characterization of *Ergasilus* species in the present study was performed using 2 partial fragments of nuclear ribosomal DNA, i.e. 18S and 28S rDNA. Moreover, these 2 nuclear markers have been also applied in previous phylogenetic studies of Ergasilidae (Song *et al*., 2008; Santacruz *et al*., 2020; Kvach *et al*., 2021). In the present study, no intraspecific genetic variability in both markers linked with host or locality was detected. However, low-intraspecific divergence in 18S and 28S rDNA was previously recorded in some representatives of ergasilid copepods (Song *et al*., 2008; Ondračková *et al*., 2019). The lake is 673 km-long, and only a relatively small part was sampled (the eastern shore of its northern end) and localities were roughly 100 km apart from each other. Therefore, a more extensive sampling comprising larger areas should be carried out to confirm genetic variability at the species level also in ergasilid species in Lake Tanganyika. Both genetic markers that were analysed differed in genetic distances (0.1% for 18S and 2% for 28S) among *Ergasilus* species in Lake Tanganyika. The 28S marker was shown to be much more efficient for species delimitation and the species found in this study were clearly resolved by means of its DNA sequence variability.

In contrast, the 18S marker was shown to be highly conservative within *Ergasilus* species found in Lake Tanganyika and almost no variability among species was observed. Previous phylogenetic studies confirmed that the 18S gene is more informative for resolving relationships between copepods at the genus and family levels (Huys *et al*., 2006; Marrone *et al*., 2013). Even though it is suitable for determining higher taxa, on the species level it does not show significant differences. This corresponds with previous studies which noted that 18S sequences are not suitable for determining the species-level richness of environmental samples, because they could underestimate species diversity (Tang *et al*., 2012) and fail to resolve relationships between closely related species (Taniguchi *et al*., 2004). Compared with the nuclear ribosomal genes, the commonly used barcoding gene, cytochrome c oxidase subunit I (COI mtDNA), has proven useful in free living copepods (Baek *et al*., 2016; Mayor *et al*., 2017). Moreover, COI data display high resolution at species level and may be also more efficient for revealing intraspecific variation (Tang *et al*., 2012). Since there are only 7 COI sequences from *Ergasilus* species [*E. jaraquensis* (Thatcher and Robertson B.A., 1982) (2 sequences), *E. wilsoni* (1 sequence) and *Ergasilus* sp. (4 sequences) published in the GenBank on 17 October 2022], the analysis of COI marker is more than suitable in future studies regarding this group of copepodes. Nevertheless, the use of Folmer's (Folmer *et al*., 1994) ‘universal’ COI primers has failed and showed to be problematic in the present study. Universal primers amplified non-ergasilid DNA pointing to contamination problems. Therefore, successful COI amplification in ergasilids will require above all the development of taxon-specific primers and to improve DNA barcoding protocols of parasitic copepods.

The phylogenetic reconstruction suggests that African ergasilids in Lake Tanganyika form a well-supported monophyletic lineage, though relationships within Ergasilidae still remain largely unresolved. This close relationship between Lake Tanganyika ergasilids may be related to the geographic origin of the species or to the endemism of their hosts. Assuming that parasite endemism correlates with the endemism of their hosts (Morand and Guégan, 2000) and assuming the existence of coevolution between parasites and their hosts, all newly described *Ergasilus* species could be considered as endemic to Lake Tanganyika. To date, at the level of parasitic crustaceans, 8 branchiuran species from the genus *Argulus* (Cunnington, 1913; Fryer, 1965, 1968; Rushton-Mellor, 1994*a*, 1994*b*), 2 species of *Lernaea* (Cunnington, 1914) and *Ergasilus flaccidus* (Fryer, 1965) collected from the endemic species *Oreochromis tanganicae* (Fryer, 1965) may also be considered as endemic species of Lake Tanganyika. The distributions of *E. megachier* and *E. macrodactylus* comprise other African areas (see below) and their host range includes also non-endemic host species. Molecular data would be particularly informative to support this hypothesis.

Overall, the relationships found within this family are consistent with previous phylogenetic analyses (Song *et al*., 2008; Santacruz *et al*., 2020; Kvach *et al*., 2021; Oliveira *et al*., 2021) and suggest that the genus *Ergasilus* is not monophyletic. Nevertheless, further morphological studies combined with molecular data are needed to elucidate the evolutionary relationships and origin of this diverse group of parasitic copepods.

## Conclusion

The present study represents the first molecular and systematic update on ergasilids from Africa since more than 3 decades. The extent of the parasitic copepod fauna of the African region is most likely underestimated and harbours a great potential for discovering new species and new host records, using a similar approach combining morphological and molecular data. This study also highlights the need for further intensive genetic research on ergasilid species in order to elucidate the phylogenetic relationships within Ergasilidae. Future studies should consider using COI marker, since 18S rDNA is not suitable for species delimitation and 28S rDNA is limited.

## Data Availability

Type and voucher specimens were deposited in the Institute of Parasitology, Czech Academy of Sciences, České Budějovice, Czech Republic (accession codes Cr-33, Cr-34, Cr-35, Cr-36 and Cr-37). The sequences produced in this study were deposited in GenBank of NCBI at https://www.ncbi.nlm.nih.gov/ (accession codes OQ407465, OQ407466, OQ407467, OQ407468, OQ407469, OQ407470, OQ407471, OQ407472, OQ407473 and OQ407474).
